# Microbiota as a state-of-the-art approach in precision medicine for pancreatic cancer management: A comprehensive systematic review

**DOI:** 10.1016/j.isci.2025.112314

**Published:** 2025-03-28

**Authors:** Zeinab Hesami, Fattaneh Sabzehali, Babak Khorsand, Samira Alipour, Amir Sadeghi, Nastaran Asri, Valerio Pazienza, Hamidreza Houri

**Affiliations:** 1 Student Research Committee, Foodborne and Waterborne Diseases Research Center, Research Institute for Gastroenterology and Liver Diseases, Shahid Beheshti University of Medical Sciences, Tehran, Iran; 2 Foodborne and Waterborne Diseases Research Center, Research Institute for Gastroenterology and Liver Diseases, Shahid Beheshti University of Medical Sciences, Tehran, Iran; 3 Department of Neurology, University of California, Irvine, Irvine, CA, USA; 4 Gastroenterology and Liver Diseases Research Center, Research Institute for Gastroenterology and Liver Diseases, Shahid Beheshti University of Medical Sciences, Tehran, Iran; 5 Celiac Disease and Gluten Related Disorders Research Center, Research Institute for Gastroenterology and Liver Diseases, Shahid Beheshti University of Medical Sciences, Tehran, Iran; 6 Division of Gastroenterology, Fondazione IRCCS Casa Sollievo della Sofferenza, San Giovanni Rotondo, Italy

**Keywords:** Microbiome, Cancer

## Abstract

Emerging evidence suggests that harnessing the microbiome holds promise for innovative diagnostic and therapeutic strategies in the management of pancreatic cancer (PC). This study aims to systematically summarize the microbial markers associated with PC and assess their potential application in clinical outcome. Forty-one studies were included to assess the associations between microbial markers and PC. Among these, 13 were developed prediction models related to the microbiome in which the highest diagnostic and prognostic model belong to blood and intratumor markers, respectively. Notably, findings that utilize microbiotas from various body sites were elucidated, demonstrating their importance as unique signatures in biomarker discovery for diverse clinical applications. This review provides unique perspectives on overcoming challenges in PC by highlighting potential microbial-related markers as non-invasive approaches. Further clinical studies should evaluate the utility and accuracy of key indicators in the microbiome as a personalized tool for managing PC.

## Highlights

• Distinct oral and gut microbiome signatures show promise as biomarkers for pancreatic cancer
• Microbiome-based models show high performance in detecting pancreatic cancer
• *Fusobacterium* and *Streptococcus* species linked to pancreatic cancer prognosis and survival
• Gut metabolites (TMAO, 3-IAA) are emerging diagnostic targets in pancreatic cancer

## Graphical abstract

**Figure:**
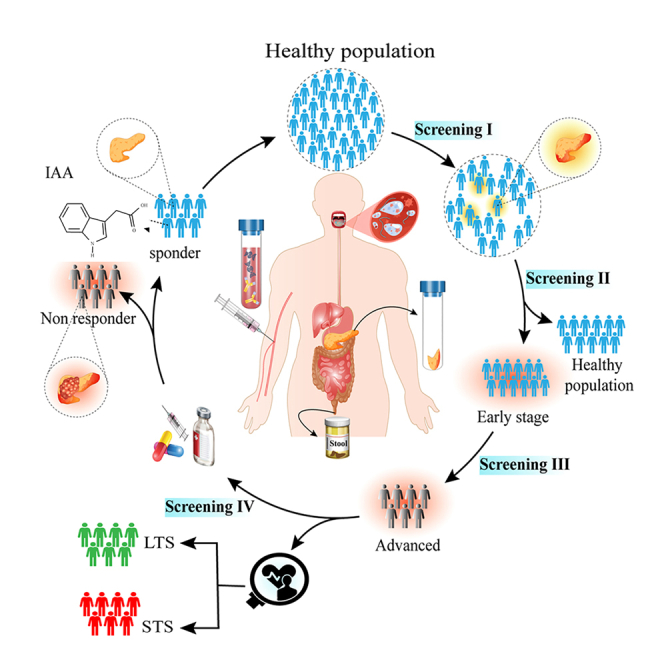

## Introduction

Pancreatic cancer (PC) is currently the seventh most lethal form of malignancy and is estimated to reach the 2^nd^ leading cause of cancer death worldwide, outstripping breast cancer by 2030.^1^^,^^2^^,^^3^ According to the National Program of Cancer Registries (NPCR) survey, which has been conducted on 65% of the US population, the incidence of PC is increasing more rapidly among younger individuals than the elderly population and also more quickly in women than in men.^4^ PC is considered a notorious malignancy for its high propensity to rapidly disseminate to both the lymphatic system and distant organs at the time of diagnosis.^5^ The intrinsic aggressive biology of PC confers resistance to the vast majority of therapies. Additionally, being asymptomatic during early stages and unspecific developed signs at advanced stages, as well as the lack of reliable biomarkers, contribute to late detection, leading to low overall survival (OS) of approximately 5 years in 5% of patients suffering from PC.^6^ Despite surgical intervention being possible in cases where the tumor can be resectable, only near to 35% of eligible patients manage to survive beyond 5 years.^7^

Despite the obscure etiology of the disease, various risk factors, including age, smoking, type 2 diabetes, obesity, and chronic pancreatitis, have been suggested to be associated with PC.^8^^,^^9^^,^^10^^,^^11^^,^^12^^,^^13^ The presence of these factors has been linked to host-microbial dysbiosis, which may have implications for the risk of developing PC.^14^^,^^15^^,^^16^^,^^17^^,^^18^^,^^19^^,^^20^ Moreover, among investigated risk factors, chronic pancreatitis (CP) is strongly related to a markedly elevated susceptibility to PC, indicating that low-grade inflammation might be implicated in either pathogenesis or progression of the disease.^21^ Inflammation can augment cellular proliferation and mutagenesis, diminish adaptability to oxidative stress, promote angiogenesis, inhibit apoptosis, and increase the secretion of inflammatory mediators.^22^ Interestingly, the human microbiome plays a pivotal role in inducing inflammation and triggering the immune system response, hence potentially contributing to the progression of PC.^23^ Consequently, given the failures and heterogeneity observed in response to the standard therapeutic options for PC, it is crucial to employ more innovative and precise approaches to holistically manage the disease.

The human body harbors an extensive diversity of symbiotic microorganisms, known as microbiota, which are estimated to exceed 100 trillion in number and are approximately equivalent to the amount of human cells.^24^ The microbiome, which refers to the collective genetic material of microbiota, surpasses the human genomes in terms of quantity.^25^ Although the microbiota of the human body is primarily concentrated in the gastrointestinal tract, it can also be found on both internal and external surfaces.^26^ Numerous studies have demonstrated a correlation between the progression of PC and the microbes residing within the oral cavity, blood, duodenal fluid, gut, and tumor microenvironment.^27^^,^^28^^,^^29^^,^^30^ According to the results of analysis, it is frequently observed that the outcome of the disease has directly associated with microbial diversity. It has been hypothesized that gut constitutes the origin of bacteria reported in pancreatic tissue, thus shaping the intratumoral bacterial niche.^31^^,^^32^ Since specific bacterial species within the microbiota are capable of stimulating inflammatory responses, mounting evidence indicates that it may be linked to precancerous conditions of the pancreas, such as CP and intraductal papillary mucinous neoplasm (IPMN).^33^^,^^34^ Also, the study of dysbiosis in microbial community in different body sites has recently catapulted interest in building prediction models with high accuracy in order to address the obstacles encountered in PC.^27^^,^^28^^,^^31^ Intriguingly, the observed uniqueness composition of microbiota in each individual has the potential to serve as a powerful tool for diagnosis in early stages, disease prognosis and screening, as well as estimation of OS and resistance status among patients who have undergone chemotherapy. In this review, we aim to summarize the latest approaches based on microbiome to investigate the possible applications of this field in personalized medicine for control and management of PC.

## Results

### Summary of selected studies

**Figure 1:**
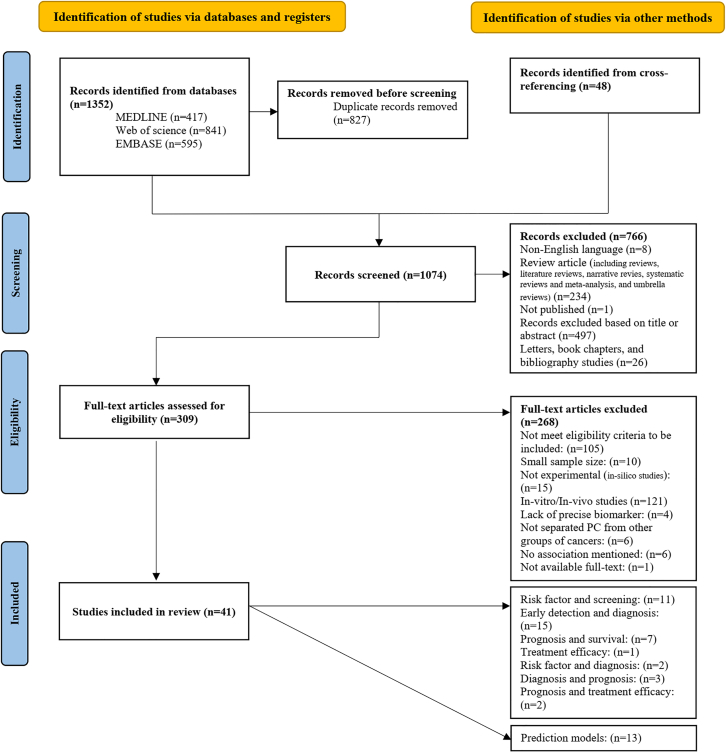
PRISMA flow diagram of the selection of studies

A total of 1,352 records were retrieved, 417 from MEDLINE, 841 from Web of Science, and 595 from EMBASE. After removing duplicates as well as adding cross-referenced studies, the first screening step was performed, and 1,074 unique records were screened, of which 309 were evaluated for eligibility through full-text screening. Following a parallel review in the final screening phase, 41 articles met the eligibility criteria and were included in this systematic review. The included studies were categorized into four main sections based on their focus on microbiome assessments: (1) 11 studies evaluated the risk factors and screened individuals at high risk of developing PC^35^^,^^36^^,^^37^^,^^38^^,^^39^^,^^40^^,^^41^^,^^42^^,^^43^^,^^44^^,^^45^; (2) 15 studies focused on the early detection and diagnosis of PC^27^^,^^28^^,^^34^^,^^46^^,^^47^^,^^48^^,^^49^^,^^50^^,^^51^^,^^52^^,^^53^^,^^54^^,^^55^^,^^56^^,^^57^; (3) seven studies investigated the prognosis and survival rates of PC patients^31^^,^^58^^,^^59^^,^^60^^,^^61^^,^^62^^,^^63^; and (4) one study assessed the efficacy of treatment for PC.^64^ Notably, some studies fell into multiple categories: two studies addressed both risk factor and diagnosis,^65^^,^^66^ three studies covered diagnosis and prognosis,^29^^,^^67^^,^^68^ and two studies focused on prognosis and treatment efficacy^69^^,^^70^ (Table 1). Moreover, among the included studies, 13 developed prediction models primarily for diagnosis and prognosis and were thus classified separately (Table 2). Figure 1 represents a comprehensive overview of the study selection process. Quality assessment using the NOS criteria classified 58.5% of included studies as high quality (NOS score: 8–9), and 41.5% as moderate quality (NOS score: 6–7). More details of the NOS assessment are presented in Table S2.

**Table 1:** Summary of study characteristics

Author, year	Study region	Participant region	Study design	Sample type of interest	Groups of interest	Clinical outcome	Treatment under study	Analytical method	Marker-gene analysis	Sequencing platform (region)	Target region	Computational pipeline	Database	Visual confirmation
Farrell et al.^46^	USA	USA	Case-control	Saliva	28 PC, 27 CP, 55 non-cancer, 28 HC	Diagnosis	–	qPCR	NA	NA	NA	NA	NA	–
Riquelme et al.^31^	USA	USA	Prospective cohort	Tissue	57 LTS PDAC, 54 STS PDAC	Prognosis	–	16srRNA sequencing	OTU	Illumina MiSeq	V4	FLASH; QIIME; Trimmomatic; UCLUST; Bowtie2; RDP	SILVA	FISH
Fan et al.^45^	USA	USA	Cohort (nested case-control)	Oral mouthwash	361 PC, 371 non-cancer	Risk factor	–	16srRNA sequencing	OTU	Roche 454 FLX Titanium	V3-V4	QIIME; ChimeraSlayer	HOMD	–
Alkharaan et al.^47^	Sweden	Sweden	Case-control	Plasma	46 HGD-IPMN + cancer, 45 LGD-IPMN, 18 non-IPMN	Diagnosis	–	qPCR	NA	NA	NA	NA	NA	–
				Saliva	21 HGD-IPMN + cancer, 25 LGD-IPMN, 19 non-IPMN		–	qPCR						
Guo et al.^62^	China	China	Case-control	Tissue	17 basal-like PDAC, 23 hybrid, 22 classical PDAC	Prognosis	–	Metagenomic sequencing	*de novo*	Illumina	–	Kraken2; Bowtie2	RefSeq	FISH
Kartal et al.^27^	Spain	Spain	Case-control	Saliva, stool, tissue	57 PDAC, 29 CP, 50 non-cancer	Diagnosis s	–	16srRNA sequencing	OUT	Illumina MiSeq	V4	DADA2; MAPseq	NR	FISH
								16srRNA sequencing	ASV	Illumina MiSeq	V4	DADA2; MAPseq	NR	
								Metagenomic sequencing	mOTU	Illumina HiSeq	–	NGLess; mOTU	proGenomes2	
	Germany	Germany	Case-control	Stool	44 PDAC, 32 non-cancer	Diagnosis	–	Metagenomic sequencing	mOTU	Illumina HiSeq	–	NGLess; mOTU	proGenomes2	
Chen et al.^49^	China	China	Case-control	Saliva, stool	40 PC, 15 CP, 39 HC	Diagnosis	–	16srRNA sequencing	OTU	Illumina MiSeq	V3-V4	UPARSE; USEARCH	RDP	–
Half et al.^51^	Israel	Israel	Case-control	Stool	30 PC, 16 NAFLD, 13 HC	Diagnosis	–	16srRNA sequencing	OTU	Illumina MiSeq	V3-V4	QIIME; UPARSE; VSEARCH; UCHIME; UCLUST	SILVA; GOLD.fa	–
Torres et al.^48^	USA	USA	Case-control	Saliva	8 PC, 22 HC	Diagnosis	–	16srRNA sequencing	OTU	Illumina MiSeq	NA	QIIME; UCLUST; PyNast; RDP	GREENGENES	–
Lu et al.^50^	China	China	Case-control	Tongue coat	30 PHC, 25 HC	Diagnosis	–	16srRNA sequencing	OTU	Illumina MiSeq	V3-V4	UPARSE; FLASH; UPARSE	SILVA	–
Vogtmann et al.^44^	USA	Iran	Case-control	Saliva	273 PDAC, 285 non-cancer	Risk factor	–	16srRNA sequencing	ASV	Illumina MiSeq	V4	DADA2; QIIME2	HOMD	–
Wei et al.^43^	China	China	Case-control	Saliva	41 PDAC, 69 HC	Risk factor	–	16srRNA sequencing	OTU	Illumina NovaSeq	V3-V4	QIIME; FLASH; UCHIME; UPARSE	GOLD.fa	–
Kim et al.^28^	Korea	Korea	Case-control	Serum	38 PC, 52 HC	Diagnosis	–	16srRNA sequencing	OTU	Illumina MiSeq	V3-V4	QIIME; UCLUST	GREENGENES	–
Zhou et al.^52^	China	China	Case-control	Stool	32 PDAC, 32 HC	Diagnosis	–	Metagenomic sequencing	*de novo*	Illumina HiSeq	–	SOAPdenovo; MetaPhlAn2	NR	–
Kohi et al.^29^	USA	USA	Case-control	Duodenal fluid	74 PDAC (53 STS, 17 LTS), 98 pancreatic cyst, 134 non-cancer	Diagnosis, Prognosis	–	16srRNA sequencing	ASV	Illumina MiSeq	NR	DADA2; QIIME2	SILVA	–
					74 PDAC (53 STS, 17 LTS), 98 pancreatic cyst, 134 non-cancer	Diagnosis		18srRNA sequencing	ASV	Illumina MiSeq	NR	DADA2; QIIME2	UNITE	
Negata et al.^68^	Japan	Japan	Case-control & prospective cohort	Saliva, Stool	150 IPMN, 47 PDAC (20 early-stage, 27 advanced-stage), 235 non-cancer	Diagnosis, prognosis	–	Metagenomic sequencing	mOTU	Illumina HiSeq	–	MEGAHIT; mOTU; Bowtie2	eggNOG	–
Yang et al.^53^	China	China	Retrospective cohort	Stool	44 PC, 50 HC	Diagnosis	–	16srRNA sequencing	ASV	Illumina	V3-V4	QIIME2	GREENGENES	–
Ren et al.^67^	China	China	Case-control	Stool	85 PC (54 PHC, 31 PBTC), 57 HC	Diagnosis, Prognosis	–	16srRNA sequencing	OTU	Illumina MiSeq	V3-V5	UPARSE; RDP	GOLD.fa	–
Gaiser et al.^34^	Sweden	Sweden	Case-control	Cyst fluid	57 IPMN, 27 PC, 21 non-IPMN	Diagnosis	–	qPCR	NA	NA	NA	NA	NA	–
								16srRNA sequencing	OTU	Pacific Biosciences	V1-V8	UCHIME; RDP	HOMD	
Matsukawa et al.^54^	Japan	Japan	Prospective cohort	Stool, tissue	24 PC, 18 HC	Diagnosis	–	Metagenomic sequencing	NR	Illumina MiSeq	–	HUMAnN2; MacQIIME; Bowtie2; MetaPhlAn2	NR	–
Del Castillo et al.^35^	USA	USA	Case-control	Tissue	38 PC CT, 34 NT	Risk factor	–	16srRNA sequencing	OTU	Illumina MiSeq	V3-V4	QIIME	HOMD; GREENGENES GOLD; RefSeq	–
Huang et al.^36^	USA	China	Cohort (nested case-control)	Serum	129 PC, 285 non-cancer	Risk factor	–	LC-MS/MS	NA	NA	NA	NA	NA	–
		Singapore	Cohort (nested case-control)	Serum	58 PC, 104 non-cancer			LC-MS/MS						
Guenther et al.^70^	USA	USA	Prospective cohort	Tissue	230 GC-based PDAC, 146 non-GC-based PDAC	Prognosis, treatment efficacy	Gemcitabine	16srRNA sequencing	OTU	Illumina MiSeq	V4	RDP	SILVA	–
Guo et al.^61^	China	China	Case-control	Stool	36 Unresectable PDAC, 36 Resectable PDAC	Prognosis	–	16srRNA sequencing	OTU	Illumina MiSeq	NR	QIIME	HMDB; METLIN	–
Kharofa et al.^60^	USA	USA	Prospective cohort	Stool	16 LTS PDAC, 8 PDAC after therapy	Prognosis	–	Metagenomic sequencing	NR	Illumina NovaSeq	–	Kraken2	RefSeq	–
Mitsuhashi et al.^59^	Japan	Japan	Prospective cohort	Tissue	283 PDAC	Prognosis	–	qPCR	NA	NA	NA	NA	NA	–
Weniger et al.^69^	USA	USA	Prospective cohort	Bile	211 BRPC or LAPC	Prognosis, treatment efficacy	Gemcitabine	Culturing	NA	NA	NA	NA	NA	–
Sun et al.^65^	China	China	Case-control	Saliva	10 PC, 10 HC	Risk factor, diagnosis	–	16srRNA sequencing	OTU	Illumina MiSeq	V3-V4	QIIME; RDP; USEARCH	RDP	–
Stolzenberg-Solomon et al.^40^	USA	USA	Cohort (nested case-control)	Serum	121 PC, 226 non-cancer	Risk factor	–	ELISA	NA	NA	NA	NA	NA	–
Michaud et al.^39^	USA	European population	Cohort (nested case-control)	Plasma	405 PC, 416 non-cancer	Risk factor	–	Immunoblot array	NA	NA	NA	NA	NA	–
Petrick et al.^37^	USA	USA	Cohort (nested case-control)	Oral mouthwash	122 PC, 354 non-cancer	Risk factor	–	Metagenomic sequencing	NR	Illumina HiSeq	–	MetaPhlAn2; HUMAnN2	NR	–
Risch et al.^38^	USA	China	Case-control	Plasma	761 PC, 794 non-cancer	Risk factor	–	ELISA	NA	NA	NA	NA	NA	–
Jeong et al.^57^	Korea	Korea	Case-control	Extracellular vesicle-derived from tissue	15 PDAC CT, 15 PDAC NT	Diagnosis	–	16srRNA sequencing	OTU	Illumina MiSeq	V3-V4	QIIME; VSEARCH; UCLUST; CASPER	SILVA	–
Yu et al.^42^	USA	USA	Cohort (nested case-control)	Serum	353 PC, 353 control	Risk factor	–	Serology assay	NA	NA	NA	NA	NA	–
Tintelnot et al.^64^	Germany	Germany	Prospective cohort	Stool	10 RmPDAC, 12 NRmPDAC	Treatment efficacy	FOLFIRINOX & GnP	Metagenomic sequencing	mOTU	Illumina	–	HUMAnN3; BBMap	UHGG	–
Abe et al.^63^	Japan	Japan	Retrospective cohort	Tissue	162 Resectable PDAC (25 STS, 27 LTS)	Prognosis	–	16S metagenomic sequencing	ASV	Illumina MiSeq	V1-V2	QIIME2; DADA2	SILVA	–
Stein-Thoeringer et al.^58^	Germany	Germany	Prospective cohort	Bile	50 PDAC	Prognosis	–	Culturing & 16srRNA sequencing	ASV	Illumina MiSeq	V3-V4	QIIME2; DADA2	GREENGENES	–
Hozaka et al.^41^	Japan	Japan	Case-control	Tissue	30 IPMN (12 INV, 18 non-INV)	Risk factor	–	16srRNA sequencing	OTU	NA	V3-V4	QIIME; USEARCH	NR	–
Li et al.^56^	China	China	Case-control	Bile	8 PC, 22 CH	Diagnosis	–	16srRNA sequencing	ASV	Ion S5TM XL	V4	QIIME2	SILVA	–
Vietsch et al.^55^	Netherlands	Netherlands	Case-control	Stool in VA	15 PDAC, 9 colon AC	Diagnosis	–	Metagenomic sequencing	*de novo*	Illumina NextSeq & Illumina HiSeq	–	NR	NR	–
Irajizad et al.^66^	USA	USA	Prospective cohort & Case-control	Serum, plasma	172 PDAC, 861 non-PDAC & 94 resectable PDAC, 42 CP, 91 HC	Risk factor, diagnosis	–	LC-MS/MS	NA	NA	NA	NA	NA	–

PC, pancreatic cancer; CP, chronic pancreatitis; HC, healthy control; PDAC, pancreatic ductal adenocarcinoma; LTS, long-term survival; STS, short-term survival; IPMN, intraductal papillary mucinous neoplasm; HGD-IPMN, high-grade dysplasia intraductal papillary mucinous neoplasm; LGD-IPMN, low-grade dysplasia intraductal papillary mucinous neoplasm; PHC, pancreatic head carcinoma; PBTC, pancreatic body and tail carcinoma; CT, cancerous tissue; NT, normal tissue; GC, gemcitabine; BRPC, borderline resectable pancreatic cancer; LAPC, locally advanced pancreatic cancer; PDAC NT, normal tissue from adjacent of cancerous tissue; RmPDAC, metastatic pancreatic ductal adenocarcinoma responder to chemotherapy; NRmPDAC, metastatic pancreatic ductal adenocarcinoma non-responder to chemotherapy; INV, invasive; non-INV, non-invasive; CH, cholelithiasis; colon AC, colon adenocarcinoma; VA, vermiform appendix; LC-MS/MS, liquid chromatography with tandem mass spectrometry; OUT, operational taxonomic unit; ASV, amplicon sequence variant; FISH, fluorescence *in situ* hybridization; NA, not applicable; NR, not reported.

**Table 2:** Microbiome-based models for early detection of pancreatic cancer, prediction of prognosis, and risk of developing disease in the future

Study	Outcome	Groups	Sample type	Microbial profiles	AUC (%, 95% CI)	Sensitivity (%)	Specificity (%)	Modeling method	Internal validation	External validation
Farrell et al.^46^	Diagnosis	28 PC vs. 28 HC	Saliva	• *Neisseria elongata*; *Streptococcus mitis*	90	96.4	82.1	Logistic regression	NR	No
	Diagnosis	28 PC vs. 27 CP		• *Granulicatella adiacens*; *Streptococcus mitis*	70	55.6	85.7			
	Diagnosis	28 PC vs. 55 non-cancer		• *Granulicatella adiacens*; *Streptococcus mitis*	68.2	85.7	52.7			
Riquelme et al.^31^	Prognosis	21 LTS PDAC vs. 22 STS PDAC (DS)	Tissue	• *Pseudoxanthomonas*	82.54	NR	NR	LASSO logistic regression	10-fold cross-validations	Yes
	Prognosis	21 LTS PDAC vs. 22 STS PDAC (DS)		• *Streptomyces*	86.62	NR	NR			
	Prognosis	21 LTS PDAC vs. 22 STS PDAC (DS)		• *Saccharopolyspora*	83.67	NR	NR			
	Prognosis	21 LTS PDAC vs. 22 STS PDAC (DS)		• *Pseudoxanthomonas*; *Streptomyces*; *Saccharopolyspora*	88.89	NR	NR			
	Prognosis	21 LTS PDAC vs. 22 STS PDAC (DS)		• *Bacillus clausii*	88.1	NR	NR			
	Prognosis	21 LTS PDAC vs. 22 STS PDAC (DS)		• *Pseudoxanthomonas*; *Streptomyces*; *Saccharopolyspora*; *Bacillus clausii*	97.51	NR	NR			
	Prognosis	36 LTS PDAC vs. 32 STS PDAC (VS)		• *Pseudoxanthomonas*	86.67	NR	NR			
	Prognosis	36 LTS PDAC vs. 32 STS PDAC (VS)		• *Streptomyces*	56.67	NR	NR			
	Prognosis	36 LTS PDAC vs. 32 STS PDAC (VS)		• *Saccharopolyspora*	70	NR	NR			
	Prognosis	36 LTS PDAC vs. 32 STS PDAC (VS)		• *Pseudoxanthomonas*; *Streptomyces*; *Saccharopolyspora*	86.67	NR	NR			
	Prognosis	36 LTS PDAC vs. 32 STS PDAC (VS)		• *Bacillus clausii*	63.33	NR	NR			
	Prognosis	36 LTS PDAC vs. 32 STS PDAC (VS)		• *Pseudoxanthomonas*; *Streptomyces*; *Saccharopolyspora*; *Bacillus clausii*	99.17	NR	NR			
Kartal et al.^27^	Diagnosis	57 PDAC vs. 50 non-cancer (DS)	Stool	• 27 species	84	NR	NR	LASSO logistic regression model	10-fold cross-validations	Yes
	Diagnosis	57 PDAC vs. 50 non-cancer (DS)		• 27 species in combination with CA19-9	94	NR	NR			
	Diagnosis	44 PDAC vs. 32 non-cancer (VS)		• 27 species	83	NR	NR			
	Diagnosis	44 PDAC vs. 32 non-cancer (VS)		• 27 species in combination with CA19-9	91	NR	NR			
Chen et al.^49^	Diagnosis	17 PC vs. 20 HC (DS)	Saliva	• 10 genera	91.6	NR	NR	Random forest	10-fold cross-validations	No
	Diagnosis	16 PC vs. 23 HC (DS)	Stool	• 10 genera	85.6	NR	NR			
Half et al.^51^	Diagnosis	30 PC vs. 13 HC & 16 NAFLD (VS)	Stool	• 14 OTUs	82.5	76.9	80	Random forest	Train-test split	Yes
	Diagnosis	Israeli validation data (*n* = 16)		• 22 OTUs	83.3	90.9	66.7			
Lu et al.^50^	Diagnosis	30 PHC vs. 25 HC (VS)	Tongue coat	• *Leptotrichia*; *Fusobacterium*; *Haemophilus*; *Porphyromonas*	80.2	77.1	78.6	Random forest	10-fold cross-validations	No
Kim et al.^28^	Diagnosis	19 PC vs. 26 HC (VS)	Serum	• *Verrucomicrobia*; *Actinobacteria*	96.6	100	84.6	Logistic regression	2-fold cross-validations	No
	Diagnosis	19 PC vs. 26 HC (VS)		• *Akkermansia*; *Ruminococcaceae UCG-014*; *Ruminiclostridium*; *Lachnospiraceae UCG-001*; *Propionibacterium*; *Sphingomonas*; *Corynebacterium*	100	100	84.62			
Zhou et al.^52^	Diagnosis	32 PDAC vs. 32 HC (VS)	Stool	• 11 species	90.74	NR	NR	Random forest	Train-test split	No
Negata, 2022	Diagnosis	47 PDAC vs. 235 non-cancer (DS)	Saliva	• 19 species	82	NR	NR	Random forest	10-fold cross-validations	Yes
	Diagnosis	43 PDAC vs. 235 non-cancer (DS)	Stool	• 30 species	78	NR	NR			
	Diagnosis	47 PDAC vs. 235 non-cancer (DS)	Saliva	• 26 OTUs	80	NR	NR			
	Diagnosis	43 PDAC vs. 235 non-cancer (DS)	Stool	• 67 OTUs	79	NR	NR			
	Diagnosis	44 PDAC vs. 32 HC (VS)	Stool	• 30 species	83	NR	NR			
	Diagnosis	57 PDAC vs. 50 HC (VS)	Stool	• 30 species	74	NR	NR			
Yang et al.^53^	Diagnosis	44 PC vs. 50 HC	Stool	• *Streptococcus1 (ASV-130672)*	92.7	NR	NR	Random forest	NR	No
	Diagnosis	44 PC vs. 50 HC		• *Streptococcus2 (ASV-46476)*	91.8	NR	NR			
	Diagnosis	44 PC vs. 50 HC		• *Streptococcus3 (ASV-80973)*	88.6	NR	NR			
	Diagnosis	44 PC vs. 50 HC		• *Streptococcus4 (ASV-48117)*	91.2	NR	NR			
	Diagnosis	44 PC vs. 50 HC		• *Streptococcus5 (ASV-75617)*	80.2	NR	NR			
	Prognosis	27 PC with liver metastasis vs. 17 PC without liver metastasis		• *Streptococcus7 (ASV-61341)*	79.6	NR	NR			
Ren et al.^67^	Diagnosis	85 PC vs. 57 HC (VS)	Stool	• 40 genera	84.2	85.9	66.7	Random forest	10-fold cross-validations	No
	Diagnosis	85 PC vs. 57 HC (VS)		• *Gemmiger*	66.3	81.2	50.9			
	Diagnosis	85 PC vs. 57 HC (VS)		• *Prevotella*	71.3	78.8	57.9			
	Prognosis	22 obstructed PHC vs. 32 non-obstructed PHC (VS)		• 10 genera	78.6	81.8	68.8			
	Prognosis	22 obstructed PHC vs. 32 non-obstructed PHC (VS)		• *Streptococcus*	66.4	NR	NR			
	Prognosis	22 Obstructed PHC vs. 32 non-obstructed PHC (VS)		• *Escherichia Shigella*	65.8	NR	NR			
	Prognosis	54 PHC vs. 31 PBTC (VS)		• *Streptococcus*	73.4	NR	NR			
Li et al.^56^	Diagnosis	8 PC vs. 22 CH	Bile	• 30 genera	96.59	NR	NR	Random forest	NR	No
				• 20 genera	97.73	NR	NR			
				• 10 genera	96.59	NR	NR			
				• 5 genera	96.59	NR	NR			
				• *Sedimentibacter*; *Microbacterium*; *Rubrobacter*	98.86	NR	NR			
Irajizad, 2023	Risk factor	172 PDAC, 861 non-PDAC	Serum	• Indoleacrylic acid; indole-derivative; TMAO	62	NR	NR	LASSO logistic regression	Train-test split	Yes
		33 PDAC, 142 non-PDAC (VS)			64	NR	NR			
		37 PDAC, 225 non-PDAC (VS)			64	NR	NR			
		37 PDAC, 225 non-PDAC (VS)		• Indoleacrylic acid; indole-derivative; TMAO + cholesterol glucuronide; galactosamine; 2-hydroxyglutarate; erythritol; glucose	79	NR	NR			
		172 PDAC, 861 non-PDAC			76	NR	NR			
		37 PDAC, 225 non-PDAC (VS)		• Indoleacrylic acid; indole-derivative; TMAO + cholesterol glucuronide; galactosamine; 2-hydroxyglutarate; erythritol; glucose + CA19-9	84	NR	NR			
		172 PDAC, 861 non-PDAC			80	NR	NR			
	Diagnosis	42 CP, 91 HC (VS)	Plasma	• Indoleacrylic acid; indole-derivative; TMAO	79	NR	NR			
		94 Resectable PDAC, 91 HC (VS)			65	NR	NR			

PC, pancreatic cancer; CP, chronic pancreatitis; HC, healthy control; PDAC, pancreatic ductal adenocarcinoma; LTS, long-term survival; STS, short-term survival; NAFLD, non-alcoholic fatty liver disease; PHC, pancreatic head carcinoma; PBTC, pancreatic body and tail carcinoma; TMAO, trimethylamine N-oxide; DS, discovery set; VS, validation set; AUC, area under the curve; OTU, operational taxonomic unit; NA, not applicable; NR, not reported.

### Summary of study characteristics

The characteristics of final eligible studies are summarized in Table 1 (with more detailed information available in the Data S1). The studies included 347 IPMN and 4,742 PC patients, with approximately 43% of the latter diagnosed specifically with pancreatic ductal adenocarcinoma (PDAC). Moreover, various cohorts served as control groups across the studies, including 508 healthy controls (HCs), 4,613 non-cancer individuals, and 113 CP patients. In the studies investigating the disease prognosis and survival, 117 patients with long-term survival (LTS) PDAC were compared with 132 short-term survival (STS) PDAC. The biosamples were collected from various body sites, including saliva, tongue coat, stool, tissue, cyst fluid, duodenal fluid, serum, plasma, and bile juice, with the most prevalent specimens being from the oral cavity and gut (Table 1).

### Methodological characteristics

Among the included studies, DNA sequencing was the primary analytical method used to directly assess microbial abundance, whereas four studies employed quantitative polymerase chain reaction (qPCR) and two utilized conventional culturing methods. Furthermore, other methods for assessing microbial-related communities indirectly included measuring antibodies against specific microbes using serology assays (three studies) and analyzing microbiota-derived metabolites using liquid chromatography with tandem mass spectrometry (LC-MS/MS) (two studies) (Table 1). In the sequencing methods used across the studies, most employed marker-gene analysis, whereas others utilized metagenomic analysis. For marker-gene analysis, the 16S ribosomal RNA (16S rRNA) gene was typically amplified, except in one study focused on fungi. The most commonly amplified hypervariable region was V3-V4. All amplicons were sequenced using high-throughput methods across five different sequencing platforms, with Illumina MiSeq being the most frequently utilized. For bioinformatic processing, the majority of studies conducting 16S rRNA sequencing used QIIME, with operational taxonomic unit (OTU) being the primary marker gene; however, heterogeneous pipelines were employed for taxonomic profiling in metagenomic analyses. Additionally, 11 reference databases were used for bacterial taxonomic assignment, with SILVA, GREENGENES, the Human Oral Microbiome Database (HOMD), and the Genomes Online Database (GOLD) being the most common in descending order.

As reported in Table 2, among the eligible studies, logistic regression was used for OR calculation, the Cox model was applied for HR calculation, and the Kaplan-Meier estimate was used for survival curves. Furthermore, other statistical analysis for relevant outcomes (microbial and microbial-related biomarkers) included the Wilcoxon rank-sum test, Kruskal-Wallis test, Kolmogorov-Smirnov test, Student’s t test, and Mann-Whitney U test. Studies that utilized receiver operating characteristic (ROC) curves employed either LASSO logistic regression or random forest models to develop prediction models (Table 3).

**Table 3:** Summary of microbial-related biomarkers

Study	Biomarker	Statistical outcome (*p* value)	Statistical test	Low survival time	High survival time	Median follow-up	Comparisons	Sample type	Clinical outcome	Treatment under study
Farrell et al.^46^	*Neisseria elongata*	FC: −2.84 (0.02)	Wilcoxon rank-sum test	–	–	–	28 PC vs. 28 HC	Saliva	Diagnosis	–
	*Streptococcus mitis*	FC: −2.45 (0.02)		–	–	–	28 PC vs. 28 HC			
	*Streptococcus mitis*	FC: −2.06 (0.01)		–	–	–	28 PC vs. 27 CP			
	*Granulicatella adiacens*	FC: 3.5 (0.04)		–	–	–	28 PC vs. 27 CP			
	*Granulicatella adiacens*	FC: 2.3 (0.02)		–	–	–	28 PC vs. 55 non-cancer			
	*Streptococcus mitis*	FC: −2.25 (0.002)		–	–	–	28 PC vs. 55 non-cancer			
Riquelme et al.^31^	*Pseudoxanthomonas*	HR: 5.885 (<0.0001)	Cox model; Kaplan-Meier estimate	1.66 (year)	10.14 (year)	–	27 LTS PDAC vs. 16 STS PDAC	Tissue	Prognosis & OS	–
	*Streptomyces*	HR: 4.572 (<0.0001)		1.66 (year)	9.66 (year)	–	25 LTS PDAC vs. 18 STS PDAC			
	*Saccharopolyspora*	HR: 13.47 (<0.0001)		1.66 (year)	10.14 (year)	–	25 LTS PDAC vs. 18 STS PDAC			
Fan et al.^45^	*Porphyromonas gingivalis*	OR: 1.6 [1.15–2.22] (0.0048)	Logistic regression model	–	–	–	361 PC vs. 371 Non cancer	Oral mouthwash	Risk factor	–
	*Aggregatibacter actinomycetemcomitans*	OR: 2.2 [1.16–4.18] (0.016)		–	–	–				
	*Fusobacteria*	OR: 0.94 [0.89–0.99] (0.014)		–	–	–				
	*Leptotrichia*	OR: 0.87 [0.79–0.95] (0.0029)		–	–	–				
Huang et al.^36^	TMAO (gut-microbiota-derived metabolite)	OR: 2.36 [1.3–4.26] (0.16)	Logistic regression model	–	–	–	187 PC vs. 362 non-cancer	Serum	Risk factor	–
	TMAO (gut-microbiota-derived metabolite)	OR: 2.81 [1.37–5.76] (0.12)		–	–	–	129 PC vs. 285 non-cancer			
	TMAO (gut-microbiota-derived metabolite)	OR: 1.42 [0.5–4.04] (0.99)		–	–	–	58 PC vs. 104 non-cancer			
Guenther et al.^70^	LPS	HR: 1.8 (0.001)	Cox model; Kaplan-Meier estimate	21.7 (month)	28.5 (month)	88.02 (months)	LPS vs. No LPS (PDAC receiving gemcitabine, *n* = 230)	Tissue	Prognosis & OS	–
Guo et al.^62^	*Acinetobacter*	NA∗ (0.037)	Wilcoxon rank-sum test	–	–	–	27 high abundance vs. 26 low abundance (resectable PDAC, *n* = 62)	Tissue	Prognosis & OS	–
	*Pseudomonas*	NA∗ (0.021)		–	–	–	14 high abundance vs. 39 low abundance (Resectable PDAC, *n* = 62)			
	*Sphingopyxis*	NA∗ (0.19)		–	–	–	24 high abundance vs. 29 low abundance (resectable PDAC, *n* = 62)			
Kharofa et al.^60^	*Faecalibacterium prausnitzii*	NA∗ (0.024)	Kaplan-Meier estimate	2.07 (years)	6.34 (years)	2.07 (years)	16 LTS PDAC vs. 8 PDAC after therapy	Stool	Prognosis & OS	–
	*Akkermansia muciniphila*	NA∗ (0.487)								
	*Saccharomyces*	NA ∗ (0.384)								
Mitsuhashi et al.^59^	*Fusobacterium*	HR:2.16 (0.023)	Cox model; Kaplan-Meier estimate	17.2 (months)	32.5 (months)	30.3 (months)	25 fusobacterium species positive vs. 258 Fusobacterium species negative (PDAC, *n* = 283)	Tissue	Prognosis & OS	–
Wei et al.^43^	*Streptococcus*	OR: 5.344 [1.282–22.282] (0.021)	Logistic regression model	–	–	–	41 PDAC vs. 69 HC	Saliva	Risk factor	–
	*Leptotrichia*	OR: 6.886 [1.423–33.337] (0.016)		–	–	–				
	*Neisseria*	OR: 0.309 [0.1–0.952] (0.041)		–	–	–				
	*Veillonella*	OR: 0.187 [0.055–0.631] (0.007)		–	–	–				
Matsukawa et al.^54^	*Bifidobacterium animalis*	HR: 1.65 (0.989)	Cox model	–	–	–	24 PC vs. 18 HC	Stool	Prognosis & OS	–
	*Collinsella aerofaciens*	HR:2.455 (0.913)		–	–	–				
	*Eubacterium ventriosum*	HR:71.33 (0.73)		–	–	–				
	*Klebsiella pneumoniae*	HR:1.117 (0.703)		–	–	–				
	*Roseburia intestinalis*	HR:62.76 (0.856)		–	–	–				
	*Streptococcus thermophilus*	HR:3.286 (0.9)		–	–	–				
Weniger et al.^69^	*Klebsiella pneumoniae*	HR: 0.68 (0.048)	Cox model; Kaplan-Meier estimate	16.5 (months)	20.7 (months)	18 (months)	73 *K. pneumoniae*-positive vs. 138 *K. pneumoniae-*negative (BRPC or LAPC, *n* = 211)	Bile	Prognosis & PFS	–
	*Klebsiella pneumoniae*	NR∗ (0.039)		15.3 (months)	26.6 (months)		*K. pneumoniae*-positive (80) vs. *K. pneumoniae*-negative (40)		Treatment efficacy	Gemcitabine
	*Klebsiella pneumoniae*	NR∗ (0.028)		Median not reached	18.8 (months)		Patients without quinolones (35) vs. patients with quinolones (38) (among *K. pneumoniae*-positive)			
Vogtmann et al.^44^	*Firmicutes*	OR: 1.01 [1–1.03] (0.02242546)	Logistic regression model	–	–	–	273 PDAC vs. 285 non-cancer	Saliva	Risk factor	–
	*Proteobacteria*	OR: 0.98 [0.96–0.99] (0.01342916)		–	–	–				
	*Gammaproteobacteria*	OR: 0.96 [0.93–0.99] (0.014656712)		–	–	–				
	*Pasteurellales*	OR: 0.95 [0.92–0.98] (0.0027175687)		–	–	–				
	*Actinomycetaceae*	OR: 1.11 [1–1.24] (0.042331788)		–	–	–				
	*Carnobacteriaceae*	OR: 0.96 [0.92–0.99] (0.008790104)		–	–	–				
	*Streptococcaceae*	OR:1.02 [1–1.03] (0.014091219)		–	–	–				
	*Pasteurellaceae*	OR: 0.95 [0.92–0.98] (0.002717569)		–	–	–				
	*Actinomyces*	OR: 1.11 [1–1.24] (0.0456839828)		–	–	–				
	*Streptococcus*	OR: 1.01 [1–1.03] (0.0207218715)		–	–	–				
	*Haemophilus*	OR: 0.95 [0.92–0.98] (0.0043243747)		–	–	–				
	*Spirochaetes*	OR: 1.48 [1.03–2.12] (0.03414989)		–	–	–				
	*Clostridia*	OR: 2.67 [1.29–5.83] (0.010085934)		–	–	–				
	*Alphaproteobacteria*	OR: 2.52 [1.28–5.28] (0.009758055)		–	–	–				
	*Spirochaetia*	OR: 1.48 [1.03–2.12] (0.034149894)		–	–	–				
	*Bifidobacteriales*	OR: 2.28 [1.3–4.11] (0.00482573)		–	–	–				
	*Clostridiales*	OR: 2.67 [1.29–5.83] (0.01008593)		–	–	–				
	*Enterobacteriales*	OR: 2.8 [1.69–4.78] (0.00009915419)		–	–	–				
	*Spirochaetales*	OR: 1.48 [1.03–2.12] (0.03414989)		–	–	–				
	*Bifidobacteriaceae*	OR: 2.28 [1.3–4.11] (0.00482573)		–	–	–				
	*Corynebacteriales*	OR: 0.5 [0.26–0.94] (0.03319118)		–	–	–				
	*Bacteroidaceae*	OR: 1.9 [1.29–2.84] (0.001377953)		–	–	–				
	*Staphylococcaceae*	OR: 1.81 [1.25–2.62] (0.001584016)		–	–	–				
	*Lactobacillales*	OR: 1.88 [1.11–3.24] (0.02045318)		–	–	–				
	*Eubacteriaceae XV*	OR: 2.22 [1.31–3.8] (0.003266748)		–	–	–				
	*Enterobacteriaceae*	OR: 2.8 [1.69–4.78] (0.00009915419)		–	–	–				
	*Spirochaetaceae*	OR: 1.48 [1.03–2.12] (0.03414989)		–	–	–				
	*Bifidobacterium*	OR: 1.49 [1.04–2.13] (0.02801301)		–	–	–				
	*Slackia*	OR: 2.1 [1.14–3.97] (0.0198536)		–	–	–				
	*Bacteroidetes [G-3]*	OR: 1.66 [1.06–2.62] (0.02737094)		–	–	–				
	*Bacteroidaceae [G-1]*	OR: 2.1 [1.38–3.22] (0.0006107645)		–	–	–				
	*Staphylococcus*	OR: 1.81 [1.25–2.62] (0.001584016)		–	–	–				
	*Clostridiales [F-1][G-2]*	OR: 2.16 [1.2–3.97] (0.0113018)		–	–	–				
	*Pseudoramibacter*	OR: 2.22 [1.31–3.8] (0.003266748)		–	–	–				
	*Lachnospiraceae [G-7]*	OR: 2.4 [1.52–3.84] (0.0002057059)		–	–	–				
	*Shuttleworthia*	OR: 2.01 [1.31–3.11] (0.001520919)		–	–	–				
	*Dialister*	OR: 1.78 [1.23–2.58] (0.0024051)		–	–	–				
	*Treponema*	OR: 1.48 [1.03–2.12] (0.03414989)		–	–	–				
Torres et al.^48^	Higher ratio of *Leptotrichia* to *Porphyromonas*	NA ∗ (<0.001)	Kruskal-Wallis test	–	–	–	8 PC vs. 22 HC	Salivary	Diagnosis	–
Negata et al.^68^	*Faecalibacterium prausnitzii*	HR: <1 (NR)	Cox model	–	–	15.3 (months)	High-risk PDAC vs. low-risk PDAC (with chemotherapy) (37)	Stool	Prognosis & OS	FOLFIRINOX/nab-paclitaxel
	*Enterobacteriaceae* sp.	HR: <1 (NR)		–	–	15.3 (months)				
	*Blautia wexlerae*	HR: <1 (NR)		–	–	15.3 (months)				
	*Clostridium* sp. *AT4*	HR: <1 (NR)		–	–	15.3 (months)				
	*Roseburia inulinivorans*	HR: <1 (NR)		–	–	15.3 (months)				
	*Parabacteroides goldsteinii*	HR: <1 (NR)		–	–	15.3 (months)				
	*Lactobacillus gasseri*	HR: >1 (NR)		–	–	15.3 (months)				
	*Ruminococcus torques*	HR: >1 (NR)		–	–	15.3 (months)				
Stolzenberg-Solomon et al.^40^	*Helicobacter pylori* seropositive	OR: 1.71 [0.97–3.01] (NR)	Logistic regression	–	–	10 (years)	121 PC vs. 226 non-cancer	Serum	Risk factor	–
	*Helicobacter pylori* with CagA+ seropositive	OR: 1.85 [1.02–3.36] (NR)		–	–	10 (years)				
	*Helicobacter pylori* seropositive	OR: 1.87 [1.05–3.34] (NR)		–	–	10 (years)				
	*Helicobacter pylori* with CagA+ seropositive	OR: 2.01 [1.09–3.7] (NR)		–	–	10 (years)				
Alkharaan et al.^47^	IgG against *Fusobacterium nucleatum*	NA ∗ (<0.0006)	Kolmogorov-Smirnov test	–	–	–	46 HGD-IPMN & IPMN with invasive cancer vs. 18 non-IPMN	Plasma	Diagnosis	–
	IgA against *Fusobacterium nucleatum*	NA ∗ (0.007)		–	–	–	25 LGD-IPMN vs. 19 Non-IPMN	Salivary		
	IgA against *Streptococcus gordonii*	NA ∗ (0.004)		–	–	–				
Michaud et al.^39^	IgG against *Porphyromonas gingivalis* (>200 ng/mL vs. ≤200 ng/mL)	OR: 2 [1–4] (NR)	Logistic regression model	–	–	–	405 PC vs. 416 non-cancer	Plasma	Risk factor	–
	IgG against *Porphyromonas gingivalis* (>200 ng/mL vs. ≤200 ng/mL)	OR: 2.14 [1.05–4.36] (NR)		–	–	–				
Petrick et al.^37^	*Porphyromonas gingivalis*	OR: 1.69 [0.8–3.56] (NR)	Logistic regression model	–	–	–	43 PC vs. 131 non-cancer (among never smoker)	Oral mouthwash	Risk factor	–
	*Prevotella intermedia*	OR: 1.4 [0.69–2.85] (NR)		–	–	–				
	*Tannerella forsythia*	OR: 1.36 [0.66–2.77] (NR)		–	–	–				
	*Ascomycota*	OR: 1.11 [0.67–1.85] (NR)		–	–	–	122 PC vs. 354 non-cancer			
	*Saccharomyces*	OR: 1.87 [0.81–4.35] (NR)		–	–	–				
	*Aggregatibacter actinomycetemcomitans*	OR: 2.33 [0.55–9.79] (NR)		–	–	–	28 PC vs. 80 non-cancer (among men)			
	*Treponema denticola*	OR: 1.73 [0.58–5.14] (NR)		–	–	–				
	*Prevotella intermedia*	OR: 1.56 [0.52–4.68] (NR)		–	–	–				
	*Filifactor alocis*	OR: 1.34 [0.53–3.41] (NR)		–	–	–				
	*Tannerella forsythia*	OR: 1.3 [0.35–4.87] (NR)		–	–	–				
Risch et al.^38^	*Helicobacter pylori* with CagA- seropositive	OR: 1.28 [0.76–2.13] (0.35)	Logistic regression model	–	–	–	761 PC vs. 794 non-cancer	Plasma	Risk factor	–
	*Helicobacter pylori* with CagA+ seropositive	OR: 0.68 [0.54–0.84] (0.00052)		–	–	–				
Jeong et al.^57^	*Tepidimonas*	FC: 2.75 (<0.005)	Student’s t test	–	–	–	15 PDAC CT vs. 15 PDAC NT	Extracellular vesicle derived from tissue	Diagnosis	–
Gaiser et al.^34^	*Fusobacterium nucleatum*	NA ∗ (<0.01)	Kruskal-Wallis test	–	–	–	4 HGD-IPMN vs. 9 non-IPMN	Cyst fluid	Diagnosis	–
	LPS	NA ∗ (<0.05)		–	–	–	24 IPMN vs. 5 non-IPMN			
	LPS	NA ∗ (<0.05)		–	–	–	5 PC vs. 5 non-IPMN			
Yu et al.^42^	*Helicobacter pylori* seropositive	OR: 0.85 [0.49–1.49] (NR)	Logistic regression model	–	–	23.9 (years) (Follow up duration)	353 PC vs. 353 control	Serum	Risk factor	–
Kohi et al.^29^	*Fusobacterium*	NA ∗ (0.046)	Student’s t test	1.45 (years)	5.1 (years)	–	34 STS PDAC vs. 17 LTS PDAC	Duodenal fluid	Prognosis & OS	–
	*Rothia*	NA ∗ (0.007)				–				
	*Neisseria*	NA ∗ (0.015)				–				
Tintelnot et al.^64^	3-IAA	NR ∗ (0.0018)	Kaplan-Meier estimate	26.4 (weeks)	51.9 (weeks)	–	10 RmPDAC vs. 12 NRmPDAC	Serum	Treatment efficacy & prognosis	FOLFIRINOX and GnP
	3-IAA	NR ∗ (0.0039)		12.8 (weeks)	40.9 (weeks)	–			Treatment efficacy, prognosis & PFS	
Abe et al.^63^	*Peptoniphilus*	HR: 4.62 (<0.001)	Cox model; Kaplan-Meier estimate	–	–	23.6 (months)	STS PDAC vs. LTS PDAC (resectable PDAC, *n* = 52)	Tissue	Prognosis & OS	–
	*Lactobacillus*	HR: 4.06 (<0.001)		–	–					
	*Bacteroides*	HR: 5.95 (<0.001)		–	–					
	*Peptoniphilus* + *Lactobacillus* + *Bacteroides*	HR: 3.48 (<0.001)		–	–		Presence of prognostic bacteria vs. absence of prognostic bacteria			
Stein-Thoeringer et al.^58^	*Enterococcus* spp.	HR: 2.51 (0.02)	Cox model; Kaplan-Meier estimate	–	–	–	STS PDAC vs. LTS PDAC (PDAC, *n* = 50)	Bile	Prognosis & OS	–
	*Klebsiella*	HR: 2.31 (0.042)		–	–	–				
	*Streptococcus anginosus*	HR: 2.8 (0.033)		–	–	–				
Hozaka et al.^41^	*Fusobacteria*	NA ∗ (0.04)	Mann-Whitney U test	–	–	–	12 INV IPMN vs. 18 non-INV IPMN	Tissue	Risk factor	–
	*Bacteroidetes*	NA ∗ (<0.01)		–	–	–				
Irajizad et al.^66^	Indoleacrylic acid; indole-derivative; TMAO	OR: 1.72 [1.25–2.37] (<0.001)	Logistic regression model	–	–	5 (years) (Follow up duration)	37 PDAC, 225 non-PDAC	Serum	Risk factor	–
	Indoleacrylic acid; indole-derivative; TMAO	OR: 1.5 [1.28–1.76] (<0.001)		–	–	5 (years) (follow-up duration)	172 PDAC, 861 non-PDAC			
	Indoleacrylic acid; indole-derivative; TMAO + cholesterol glucuronide; galactosamine; 2-hydroxyglutarate; rrythritol; glucose+CA-19-9	OR: 2.83 [1.83–4.82] (NR)		–	–	–	41 CP, 91 HC			
	Indoleacrylic acid; indole-derivative; TMAO + cholesterol glucuronide; Galactosamine; 2-hydroxyglutarate; erythritol; glucose+CA-19-9	OR: 1.55 [1.13–2.23] (NR)		–	–	–	94 resectable PDAC, 91 HC			
	Indoleacrylic acid; indole-derivative; TMAO + cholesterol glucuronide; galactosamine; 2-hydroxyglutarate; erythritol; glucose	OR: 3.13 [2.08–4.98] (<0.001)		–	–	5 (years) (follow-up duration)	37 PDAC, 225 non-PDAC			
	Indoleacrylic acid; indole-derivative; TMAO + cholesterol glucuronide; galactosamine; 2-hydroxyglutarate; erythritol; glucose	OR: 2.75 [2.25–3.38] (<0.001)		–	–	5 (years) (follow-up duration)	172 PDAC, 861 non-PDAC			
	Indoleacrylic acid; indole-derivative; TMAO	OR: 9.67 [4.56–23.3] (<0.001)		–	–	5 (years) (follow-up duration)	37 PDAC, 225 non-PDAC	Plasma	Diagnosis	
	Indoleacrylic acid; indole-derivative; TMAO	OR: 8.44 [5.8–12.2] (<0.001)					172 PDAC, 861 non-PDAC			

PC, pancreatic cancer; CP, chronic pancreatitis; HC, healthy control; PDAC, pancreatic ductal adenocarcinoma; LTS, long-term survival; STS, short-term survival; IPMN, intraductal papillary mucinous neoplasm; HGD-IPMN, high-grade dysplasia intraductal papillary mucinous neoplasm; CT, cancerous tissue; BRPC, borderline resectable pancreatic cancer; LAPC, locally advanced pancreatic cancer; PDAC NT, normal tissue from adjacent of cancerous tissue; RmPDAC, metastatic pancreatic ductal adenocarcinoma responder to chemotherapy; NRmPDAC, metastatic pancreatic ductal adenocarcinoma non-responder to chemotherapy; INV IPMN, invasive intraductal papillary mucinous neoplasm; non-INV IPMN, non-invasive intraductal papillary mucinous neoplasm; TMAO, trimethylamine N-oxide; 3-IAA, 3-indole-acetic acid; LPS, lipopolysaccharides; OS, overall survival; PFS, progression-free survival; FC, fold change; OR, odds ratio; HR, hazard ratio; NA, not applicable; NR, not reported.

### Microbial diversity

**Figure 2:**
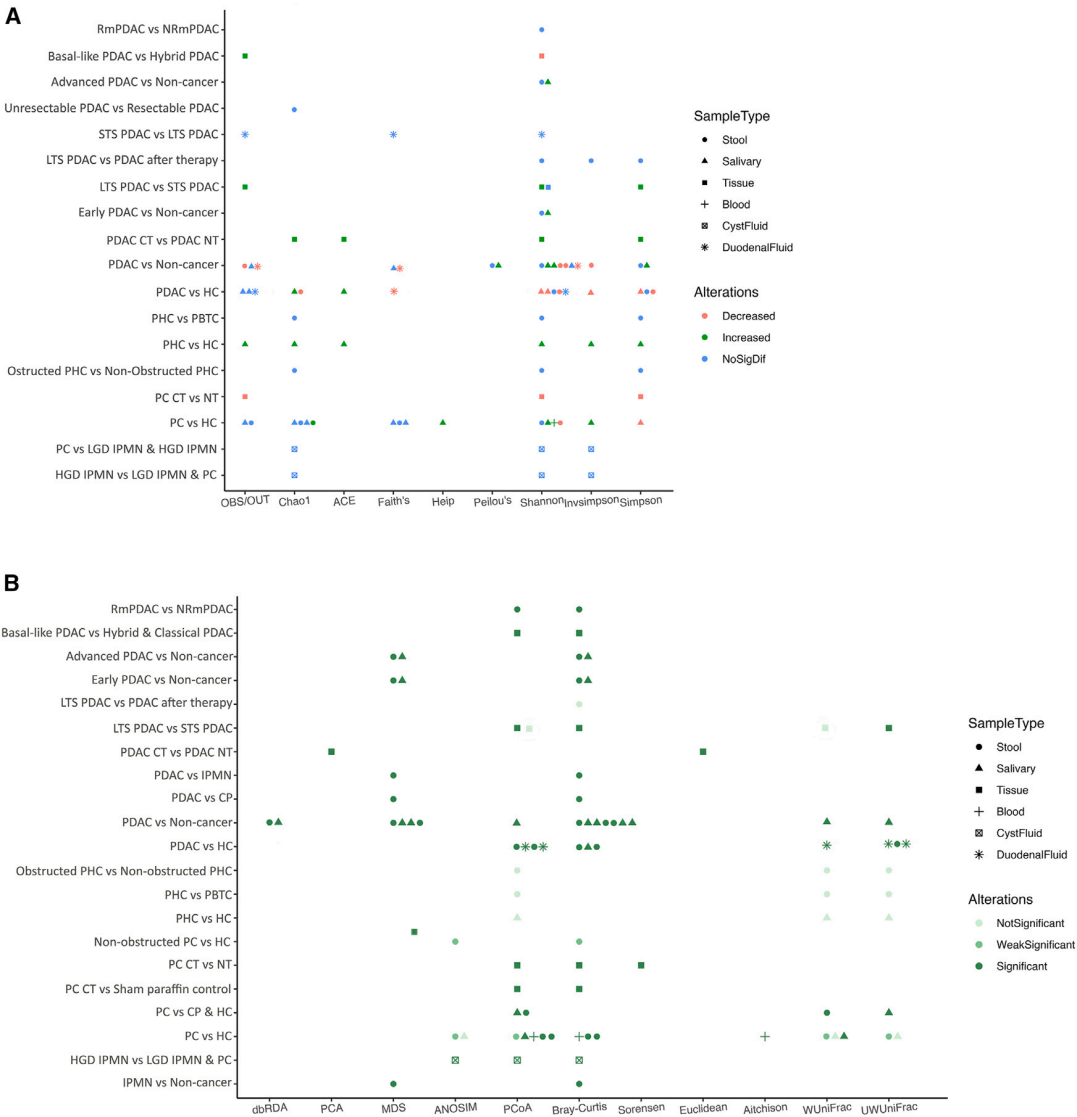
Alpha and beta diversity among different case and control groups (A) Alpha diversity. Sample types are represented with different shapes. Red indicates significantly decreased richness of diversity in cases compared to controls; green, significantly increased; and blue, no significant difference. (B) Beta diversity. Sample types are represented with different shapes. Light green indicates no significant different between cases and controls; medium green, weak significant difference; and dark green, significant difference. PC, pancreatic cancer; CP, chronic pancreatitis; HC, healthy control; PDAC, pancreatic ductal adenocarcinoma; LTS, long-term survival; STS, short-term survival; IPMN, intraductal papillary mucinous neoplasm; HGD-IPMN, high-grade dysplasia intraductal papillary mucinous neoplasm; LGD-IPMN, low-grade dysplasia intraductal papillary mucinous neoplasm; PHC, pancreatic head carcinoma; PBTC, pancreatic body and tail carcinoma; CT, cancerous tissue; NT, normal tissue; PDAC NT, normal tissue from adjacent of cancerous tissue; RmPDAC, metastatic pancreatic ductal adenocarcinoma responder to chemotherapy; NRmPDAC, metastatic pancreatic ductal adenocarcinoma non-responder to chemotherapy.

Of the included studies, 28 studies^27^^,^^28^^,^^29^^,^^31^^,^^34^^,^^35^^,^^37^^,^^41^^,^^43^^,^^44^^,^^47^^,^^48^^,^^49^^,^^50^^,^^51^^,^^52^^,^^53^^,^^55^^,^^56^^,^^57^^,^^60^^,^^61^^,^^62^^,^^63^^,^^64^^,^^65^^,^^67^^,^^68^ reported alpha diversity using various indices, including observed species/OTU, Chao 1, ACE, Faith’s, Heip, Peilou’s, Shannon, Simpson, and Invsimpson, with Simpson being the most commonly used. Most studies reported a decrease in microbial diversity in saliva, duodenal fluid, and stool samples from patients with PDAC compared to HCs or non-cancer individuals. However, salivary samples from patients with pancreatic head carcinoma (PHC) showed enriched microbial diversity as opposed to HCs. Remarkedly, salivary samples from advanced-stage PDAC patients had higher diversity than non-cancer individuals. Additionally, microbial diversity in pancreatic tissue samples from PDAC patients has been indicated to be higher in cancerous tissues compared to adjacent normal tissues. Notwithstanding, microbial diversity in pancreatic tissues from PDAC patients was lower compared to individuals without known pancreatic malignancies. Among other sample types, duodenal fluid samples exhibited no significant differences, whereas blood samples demonstrated higher microbial diversity in cases compared to controls. Notably, there were significant discrepancies in reported alpha diversity both between studies and within individual studies (Figure 2A and Data S1).

Twenty-seven studies^27^^,^^28^^,^^29^^,^^31^^,^^34^^,^^35^^,^^37^^,^^41^^,^^44^^,^^45^^,^^48^^,^^49^^,^^50^^,^^51^^,^^52^^,^^53^^,^^54^^,^^55^^,^^56^^,^^57^^,^^60^^,^^62^^,^^63^^,^^64^^,^^65^^,^^67^^,^^68^ explored beta diversity using diverse analytical methods such as dbRDA, PCA, MDS, ANOSIM, PCoA, and PERMANOVA. These studies employed indices like Bray-Curtis, Jaccard, Sorensen, Euclidean, Aitchison, Weighted UniFrac, and Unweighted UniFrac. Notably, PCoA plots and Bray-Curtis indices were particularly common. Across these analyses, most studies identified significant differences in microbial diversity between various groups of interest. However, some comparisons did not reveal significant differences, including those between PHC and pancreatic body and tail carcinoma (PBTC), PHC and HCs, obstructed PHC and non-obstructed PHC, and PDAC and colon adenocarcinoma (colon AC) (Figure 2B and Data S1).

### Alteration of microbiota

At the phylum level, studies reported findings across various body sites, including the gut, oral cavity, duodenum, tissue, and blood.^27^^,^^28^^,^^29^^,^^35^^,^^41^^,^^43^^,^^45^^,^^49^^,^^50^^,^^51^^,^^52^^,^^53^^,^^65^^,^^67^ In the gut, PC patients exhibited higher abundance of Proteobacteria and Actinobacteria, alongside lower levels of Firmicutes compared to HCs. The changes in Bacteroidetes between PC and HCs were inconsistent, with reports of both enrichment^51^^,^^52^ and depletion.^53^ In the oral cavity, PC patients showed elevated levels of Firmicutes, Verrucomicrobia, Fusobacteria, and Actinobacteria, whereas Bacteroidetes and Proteobacteria were less abundant compared to HCs.^43^^,^^44^^,^^49^^,^^50^ In the blood, PC patients displayed enrichment of Verrucomicrobia, Deferribacteres, and Bacteroidetes, whereas Actinobacteria were depleted.^28^ Additionally, in the duodenum, Fusobacteria were more abundant in PC patients compared to non-cancer individuals, with STSs showing higher levels of Fusobacteria compared to LTSs.^29^ In pancreatic cancerous tissue compared to normal tissue, Tenericutes were enriched, whereas Euryarchaeota showed depletion.^35^ Moreover, Fusobacteria and Bacteroidetes displayed higher abundance in tissues recruiting from invasive IPMN compared to non-invasive IPMN^41^ (Data S1).

**Figure 3:**
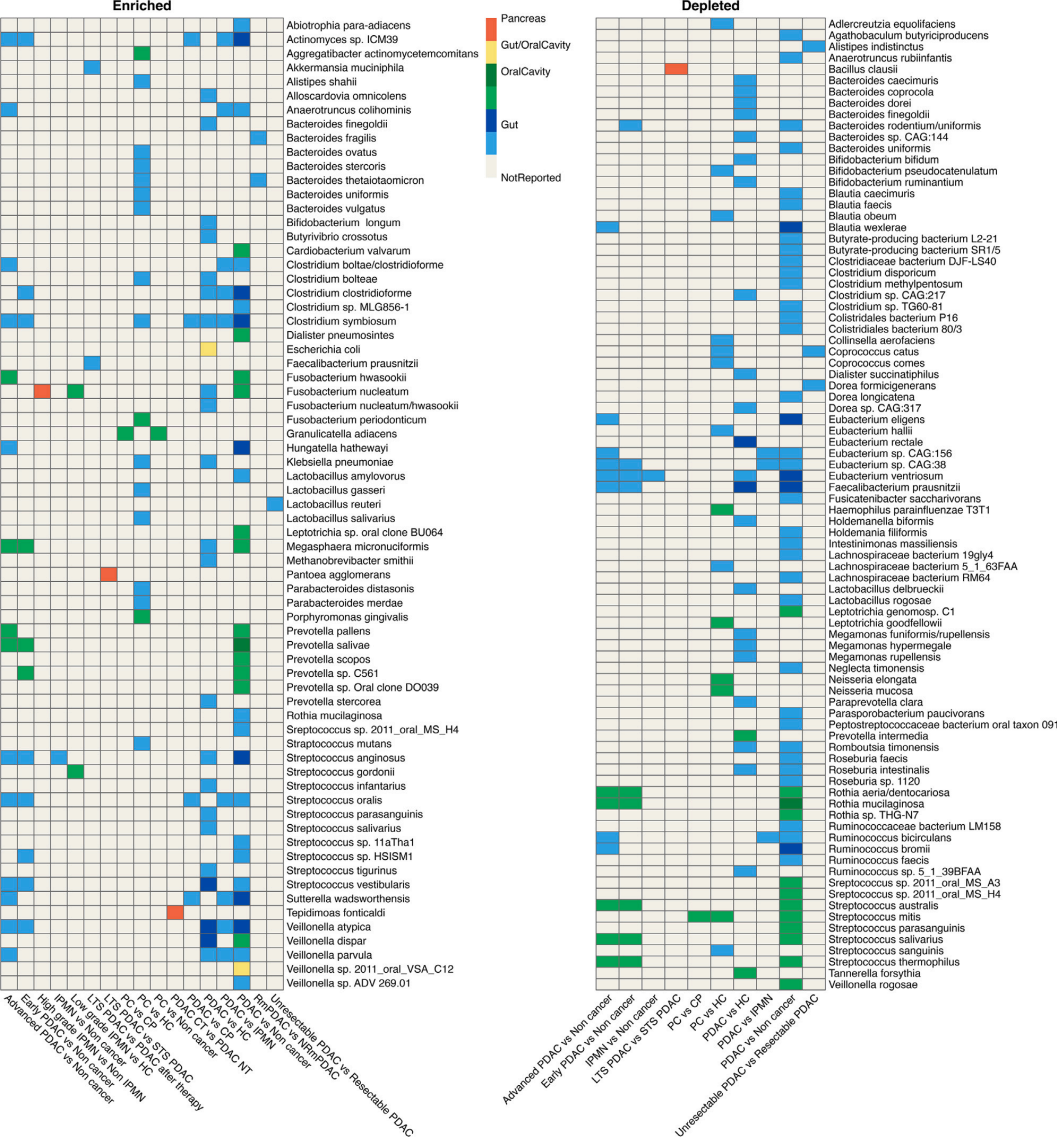
Heatmap of microbiota alterations at the species level across different groups The heatmap depicts enriched (left) and depleted (right) species in various sample types: pancreas (red), oral cavity reported in one study (light green), oral cavity reported in more than one study (dark green), gut reported in one study (light blue), gut reported in more than one study (dark blue), and species reported in both oral cavity and gut (yellow). The left heatmap depicts enriched species, whereas the right heatmap shows depleted species. PC, pancreatic cancer; CP, chronic pancreatitis; HC, healthy control; PDAC, pancreatic ductal adenocarcinoma; LTS, long-term survival; STS, short-term survival; IPMN, intraductal papillary mucinous neoplasm; HGD-IPMN, high-grade dysplasia intraductal papillary mucinous neoplasm; LGD-IPMN, low-grade dysplasia intraductal papillary mucinous neoplasm; CT, cancerous tissue; NT, normal tissue; PDAC NT, normal tissue from adjacent of cancerous tissue; RmPDAC, metastatic pancreatic ductal adenocarcinoma responder to chemotherapy; NRmPDAC, metastatic pancreatic ductal adenocarcinoma non-responder to chemotherapy.

To identify patterns in taxonomic differences at the species level among precancerous or PC states compared to controls, we visualized alterations in a heatmap (Figure 3). In PC patients, species such as *Veillonella atypica*, *Streptococcus anginosus*, and *Klebsiella pneumoniae* were enriched.^27^^,^^52^^,^^68^ In contrast, *Ruminococcus bromii*, *Faecalibacterium prausnitzii*, *Eubacterium ventriosum*, *Eubacterium rectale*, and *Blautia wexlerae* were depleted in the gut.^27^^,^^52^^,^^68^ Intriguingly, *Faecalibacterium prausnitzii* and *Akkermansia muciniphila* exhibited higher abundance in LTSs and patients cured from PC.^60^ In the oral microbiome, *Porphyromonas gingivalis* and *Aggregatibacter actinomycetemcomitans* were enriched,^45^ whereas *Streptococcus mitis*, *Rothia mucilaginosa*, *Neisseria elongata*, and *Neisseria mucosa* were depleted.^46^^,^^65^^,^^68^ Among patients with unresectable PDAC, *Lactobacillus reuteri* had higher abundance in the gut compared to those with resectable PDAC. On the other hand, *Dorea formicigenerans*, *Alistipes indistinctus*, and *Coprococcus catus* were reported to be depleted.^61^ Some species exhibited alterations across multiple body sites, such as *Fusobacterium nucleatum* in the oral cavity, gut, duodenum, and tumor.^27^^,^^34^^,^^52^^,^^68^

### Microbiota as risk factors and screening tool for developing PC

**Figure 4:**
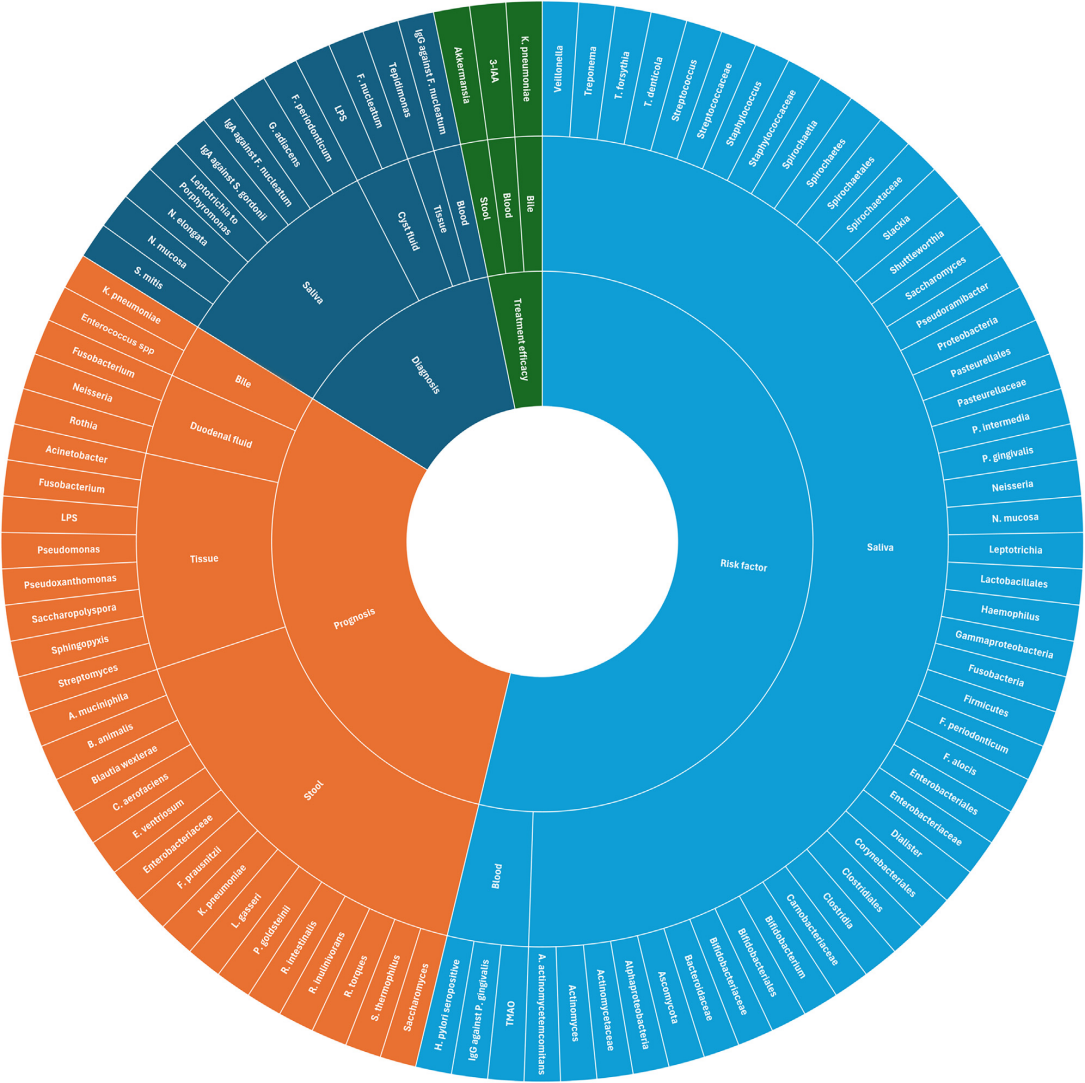
Graphical summary of microbial-related biomarkers and their potential clinical applications The figure summarizes reported biomarkers in salivary, blood, stool, tissue, bile, and duodenal fluid samples. TMAO, trimethylamine N-oxide; 3-IAA, 3-indole-acetic acid; LPS, lipopolysaccharides.

In studies examining microbial-related risk factors, biomarkers were predominantly assessed in saliva and blood samples, which were obtained from 807 to 2,036 PC patients, respectively (Table 1). Notably, dysbiosis within the oral microbiome, marked by an elevated presence of specific periodontal pathogens such as *P*. *gingivalis*,^37^^,^^45^ *Treponema denticola*,^37^ and *T*. *forsythia*^37^—collectively known as the red complex bacteria (RCB)—was implicated as a potential risk factor associated with future onset of PC (Table 3; Figure 4). Moreover, the enrichment of *A*. *actinomycetemcomitans*,^37^^,^^45^ *Fusobacterium periodonticum*,^65^ and *P*. *intermedia*^37^ has been associated with a progression toward PC. At the genus level, *Neisseria*^43^ and *Veillonella*^43^ appeared to have a protective effect, unlike *Streptococcus*^43^ and *Dialister* spp..^44^ *Leptotrichia*, however, exhibited contradictory findings; one study^45^ reported a protective effect with an odds ratio (OR) < 1, whereas another study^43^ found that carriage of *Leptotrichia* was associated with a higher risk of PDAC (OR = 6.886). Additionally, the phylum of Fusobacteria was reported to correlate with reduced risk in oral samples from PC patients compared to the non-cancer group,^45^ whereas in tissue, it was associated with the aggressiveness of IPMN.^41^ In blood samples, investigations mainly focused on microbiota-derived metabolites or antibodies against microbiota. Three serological studies^38^^,^^40^^,^^42^ explored the relationship between *Helicobacter pylori* and PC, with two studies^40^^,^^42^ reported controversial findings, whereas one study^38^ specifically examined the impact of Cag+/− *H. pylori* on the risk of developing PC (Table 3; Figure 4). Furthermore, individuals with high levels of antibodies (>200 ng/mL) against *P*. *gingivalis* had a 2-fold increased risk of PC compared to those with lower levels (≤200 ng/mL).^39^ Microbial metabolites such as indoleacrylic acid, an indole-derivative, and trimethylamine N-oxide (TMAO) were noted as potential biomarkers for identifying individuals at high risk of developing PC within 5 years.^66^

### Microbial biomarker discoveries for early detection and diagnosis

Numerous studies have described microbial biomarkers for early detection and diagnosis of PC. Several oral bacterial species have been identified as potential biomarkers, including *N*. *elongata*, *S*. *mitis*, *Granulicatella adiacens*, *N*. *mucosa*, *Leptotrichia goodfellowii*, *F*. *periodonticum*, *Prevotella salivae*, *Prevotella pallens*, *Megasphaera micronuciformis*, *Rothia aeria/dentocariosa*, *S*. *australis*, *Streptococcus thermophilus*, and *R*. *mucilaginosa*.^46^^,^^48^^,^^65^^,^^68^ Additionally, salivary antibodies against certain oral bacteria, such as elevated salivary immunoglobulin A (IgA) reactivity to *F*. *nucleatum* and *Streptococcus gordonii*, have been suggested as potential biomarkers for PC diagnosis.^47^ In the gut, several bacterial species have been recommended as potential diagnostic biomarkers, including *V*. *atypica*, *Veillonella dispar*, *Veillonella parvula*, *Streptococcus vestibularis*, *F*. *prausnitzii*, *E*. *rectale*, *Romboutsia timonensis*, *Roseburia intestinalis*, *Clostridium bolteae/clostridioforme*, *Cenarchaeum symbiosum*, *S*. *anginosus*, *Streptococcus oralis*, *Streptococcus mutans*, *Sutterella wadsworthensis*, *E*. *ventriosum*, *Ruminococcus bicirculans*, *R*. *bromii*, *Bacteroides rodentium/uniformis*, *Bacteroides thetaiotaomicron*, *Lactobacillus gasseri*, *Lactobacillus salivarius*, *Escherichia coli*, *Bifidobacterium longum*, *Bacteroides finegoldii*, *Alloscardovia omnicolens*, *Methanobrevibacter smithii*, and *Klebsiella pneumoniae*.^27^^,^^52^^,^^54^^,^^68^ However, some fecal biomarkers, such as *E*. *ventriosum*, *K*. *pneumoniae*, *R*. *intestinalis*, and *S*. *thermophilus*, did not exhibit significant differences after adjusting for multiple factors in a single study.^54^

Importantly, some species like *Streptococcus salivarius*, *Streptococcus parasanguinis*, and *F*. *nucleatum/hwasookii* were suggested as potential biomarkers in both salivary and fecal samples.^27^^,^^68^ Furthermore, several genera, including *Veillonella*, *Peptostreptococcus*, *Escherichia-Shigella*, *Streptococcus*, *Fusobacterium*, *Neisseria*, and *Haemophilus*, have been identified as biomarkers across the oral cavity, gut lumen, and duodenal fluid, leading to enhanced utility in early detection and diagnostic applications.^27^^,^^29^^,^^48^^,^^49^^,^^50^^,^^52^^,^^53^^,^^65^^,^^67^^,^^68^ According to one study on the fungal community in duodenal fluid, *Nakaseomyces* and *Skeletocutis* were investigated to differentiate between patients with PDAC and non-cancer individuals^29^ (Figures 3 and 4; Tables 3 and Data S1).

In the pancreas, elevated bacterial lipopolysaccharides (LPS) have been noted in pre-cancerous and cancerous conditions compared to non-cancerous status.^34^ Genera such as *Granulicatella*, *Lactobacillus*, *Fusobacterium*, and *Streptococcus*, found in various sites of the gastrointestinal (GI) tract,^27^^,^^29^^,^^48^^,^^49^^,^^50^^,^^53^^,^^54^^,^^65^^,^^67^ were also observed in tissue samples.^27^^,^^34^^,^^57^ In the analysis of bacteria-derived extracellular vesicles isolated from blood, genera such as *Turicibacter*, *Ruminiclostridium*, *Stenotrophomonas*, *Sphingomonas*, *Propionibacterium*, and *Corynebacterium* have been identified as potential biomarkers distinguishing between patients with PC and HCs.^28^ Additionally, circulating IgG reactivity with *F*. *nucleatum* and microbiota-derived metabolites, including indoleacrylic acid, indole derivatives, and TMAO, have been reported to hold diagnostic value.^47^^,^^66^ Notably, *Akkermansia* was the only genus reported in the oral, gut, pancreas, and extracellular vesicles acquired from blood, highlighting its potential significance in diagnostic and clinical applications^27^^,^^28^^,^^49^^,^^51^^,^^57^^,^^67^ (Figures 3 and 4; Tables 3 and Data S1).

It is worth noting that some biomarkers have been reported to distinguish PC from other diseases like colon AC and cholelithiasis (CH). In the vermiform appendix microbiome, identified in stool samples, genera such as *K*. *pneumoniae*, *Bifidobacterium animalis*, *Adlercreutzia equolifaciens*, *Waltera intestinalis*, *Bacteroides stercoris*, *R*. *bromii*, and *Barnesiella intestinihominis* were distinct in PDAC patients compared to those with colon AC.^55^ In the biliary microbiome, genera such as *Gaiella*, *Massalia*, *Nitrospira*, *Rubellimicrobium*, *Brevundimonas*, *Corynebacterium*, *Geobacter*, *Shewanella*, *Cloacibacterium*, *Cutibacterium*, *Bacillus*, *Cupriavidus*, *Staphylococcus*, *Halomonas*, *Exiguobacterium*, *Acinetobacter*, *Pseudomonas*, *Faecalibaculum*, and *Parasutterella* were reported to distinguish PC patients from those with CH^56^ (Data S1).

### Microbial signature discoveries for prognosis and survival evaluation

Intratumor bacteria might be linked to cancer cell aggressiveness, with increased levels of *Acinetobacter*, *Pseudomonas*, and *Sphingopyxis* strongly associated with carcinogenesis.^62^ Furthermore, the presence of *Fusobacterium* species in tumor niche and duodenal fluid has been independently associated with poor prognosis in PC, indicating that *Fusobacterium* could serve as a prognostic biomarker for PC.^29^^,^^59^ A comprehensive study on PDAC patients undergoing adjuvant gemcitabine (aGC) therapy found that the presence of LPS in tumors was associated with worse OS, increasing the risk by 1.8-fold compared to tissues without detectable LPS.^70^ Examination of 211 patients revealed that an increasing number of pathobiont strains, such as *K*. *pneumoniae* identified in intraoperative bile cultures, correlated with decreased progression-free survival (PFS).^69^ In the studies comparing LTS and STS in PDAC, microbiome patterns in bile, tissue, and duodenal fluid samples were investigated. Accordingly, patients with a higher abundance of *Pseudoxanthomonas*, *Streptomyces*, and *Saccharopolyspora* demonstrated better outcomes, with hazard ratio (HR) of 5.885, 4.572, and 13.47, respectively.^31^ Conversely, elevated levels of biliary *Enterococcus* spp., *Klebsiella*, *S*. *anginosus*, and duodenal *Rothia* and *Neisseria* were associated with poorer outcomes.^29^^,^^58^ Several gut species were identified as protective factors, more prevalent in patients with lower risk or longer survival. These included *F*. *prausnitzii*, *Akkermansia muciniphila*, *B*. *wexlerae*, *Roseburia inulinivorans*, *Parabacteroides goldsteinii*, *L*. *gasseri*, *Ruminococcus torques*, *Alistipes indistinctus*, *Coprococcus catus*, and *Dorea formicigenerans*^60^^,^^61^^,^^68^ (Table 3; Figure 4).

### Association between microbiota and treatment efficacy

The importance of the microbiome in modulating the efficacy of cancer treatments has been increasingly highlighted by recent studies.^71^^,^^72^^,^^73^^,^^74^^,^^75^^,^^76^^,^^77^ Notably, it has been described that PFS was significantly (*p* = 0.039) improved by aGC therapy in patients who were tested negative for *K*. *pneumoniae* based on bile culture (26.2 months) compared to those who were tested positive (15.3 months) (Table 3; Figure 4). These findings suggest that *K*. *pneumoniae* may promote chemoresistance to aGC.^69^ Similarly, it has been hypothesized that an LPS-positive tumor microbiome serves as a negative predictor of aGC efficacy^70^ (Table 3). Additionally, recent investigation revealed that the microbiota-derived tryptophan metabolite indole-3-acetic acid (3-IAA) was elevated in serum of patients who respond to FOLFIRINOX and GnP (Table 3; Figure 3). Based on metagenomic sequencing data, the class of *Gammaproteobacteria* was enriched in patients with metastatic PDAC who were non-responders to chemotherapy (NRmPDAC) compared to those who were responders (RmPDAC). Moreover, the 3-IAA-producing bacteria *Bacteroides fragilis* and *B*. *thetaiotaomicron* were found to have higher abundance in stool of responders compared to non-responders both before and during chemotherapy treatment (Data S1).^64^

### Prediction model development

**Figure 5:**
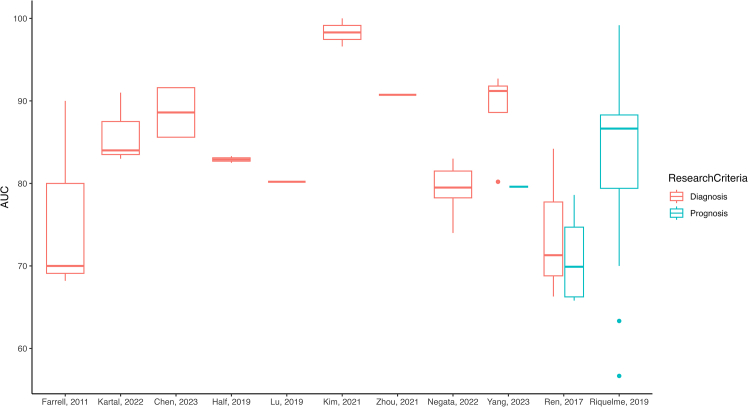
Model discriminative performance of the AUCs (area under the curves) based on microbiome signature for diagnostic (red) and prognostic (blue) outcomes

For the prediction model, 13 articles were identified that utilized microbial biomarkers for prognosis or diagnosis, except for one article that used microbial-derived metabolites for risk prediction,^66^ with six of these studies including external validation.^27^^,^^31^^,^^49^^,^^51^^,^^66^^,^^68^ A summary of the study characteristics is presented in Table 2. As depicted in Figure 5, the highest performance was achieved by a diagnostic model based on circulating biomarkers in blood using 7 OTUs, with an area under the curve (AUC) of 1.0.^28^ Among the prognostic models, *Pseudoxanthomonas*, *Streptomyces*, *Saccharopolyspora*, and *Bacillus clausii* had the highest AUC, with accuracies of 97.51% and 99.17% in the discovery and external validation sets, respectively.^31^ All oral biomarkers were used to develop diagnostic models,^46^^,^^49^^,^^50^^,^^68^ with two panels of biomarkers, one^49^ including 10 microbial OTUs and the other^46^ including *N*. *elongata* and *S*. *mitis*, achieving AUCs of 0.916 and 0.9, respectively. The majority of studies were conducted on stool samples,^27^^,^^49^^,^^51^^,^^52^^,^^53^^,^^67^^,^^68^ investigating various microbial signatures ranging from 67 OTUs^68^ to 1 OTU/ASV^53^^,^^67^ for diagnostic purposes. Among these gut studies, *Streptococcus* was identified as a potential prognostic biomarker,^53^^,^^67^ with one study distinguishing between PC patients with liver metastasis and those without, achieving an AUC of 79.6%.^53^ On the other hand, only one study employed the biliary microbiome to differentiate between PC and CH, with the highest performing models involving *Sedimentibacter*, *Microbacterium*, and *Rubrobacter* (AUC = 0.9886).^56^ Intriguingly, two studies combined biomarkers with levels of carbohydrate antigen (CA) 19-9, which improved the performance of the models in both cases.^27^^,^^66^

## Discussion

In this systematic review, we summarized the current literature on the application of the microbiome in improving clinical outcomes for PC. We identified 41 eligible studies, 13 of which developed prediction models based on microbial-related biomarkers. However, considerable heterogeneity in cohort sizes, demographic characteristics, microbiome sequencing analyses, and statistical tests prevented meta-analysis. Consequently, we have systematically highlighted key themes in the literature that may serve as future biomarkers, using the microbiome as a tool for screening individuals at high risk of developing PC, early detection of patients at early stages of the disease, predicting disease prognosis, and assessing treatment efficacy.

Among studies exploring microbial biomarkers prior to the progression of PC, two main patterns have been observed through altered oral microbiome and antibody responses to dysbiosis. The increased abundance of RCB and other periodontal pathogens, such as *A*. *actinomycetemcomitans* and *P*. *intermedia*, has been investigated for their potential roles in various gastrointestinal cancers, including colorectal cancer (CRC) and esophageal cancer (EC).^78^^,^^79^ Most importantly, *P*. *gingivalis* is recognized as a key pathogen contributing to the imbalance of the oral microbiome; both its elevated levels and the presence of circulating antibodies against it have been linked to the future onset of PC.^37^^,^^39^^,^^45^ The hypothesis underlying these observations in prospective investigations suggests that the ability of *P*. *gingivalis* to trigger inflammatory responses is a significant factor.^80^ Additionally, the secretion of *Porphyromonas* peptidylarginine deiminase (PPAD) has been suggested to be related to prevalent point mutations in Kras and p53 in PC, through the degradation of arginine.^81^^,^^82^

Findings from observational studies indicate a potential core set of oral and gut bacteria that could serve as predictive biomarkers for the diagnosis and prognosis of PC. The performance of microbial-based models varied, with AUC levels ranging from 0.5667 to 0.91 in validation sets. Remarkably, a circulating microbial panel consisting of seven OTUs achieved an AUC of 1.0 in discerning patients with PC from HCs.^28^ However, the study’s limitations include the absence of external validation sets and a small sample size. Based on observations, certain oral species such as *N*. *elongata* and *S*. *mitis*, as well as the ratio of *Leptotrichia* to *Porphyromonas*, appear promising for diagnostic purposes.^46^^,^^48^ In the realm of gut biomarkers, Veillonella spp. stand out as critical indicators, alongside established probiotic species like *F*. *prausnitzii*, *B*. *longum*, and *L*. *gasseri*.^27^^,^^43^^,^^51^^,^^52^^,^^60^^,^^67^^,^^68^ Notably, one of the most essential species reported in various studies within the oral cavity and lower GI tract was *F*. *nucleatum*.^27^^,^^34^^,^^52^^,^^68^ Originally considered a commensal, this microorganism can shift into a pathogen associated with several GI disorders, particularly contributing to the progression of CRC.^83^ Importantly, it has been demonstrated as an effective early warning and prognostic marker for CRC, highlighting its potential as a target for preventive and therapeutic interventions.^84^ Likewise, it exhibits promising diagnostic capabilities as an oral and gut biomarker in PC, with its involvement in antibody production further underscoring its diagnostic potential.^27^^,^^47^^,^^68^ To predict survival time in patients suffering from PC, three studies developed prognostic models based on features derived from fecal and tumor microbiomes.^31^^,^^53^^,^^67^ The most significant microbial predictors identified were *Pseudoxanthomonas*, *Streptomyces*, *Saccharopolyspora*, and *B. clausii*, likely due to colonization by the gut microbiome in the tumor milieu. The distinct tumor microbiome in LTS might help create a favorable tumor microenvironment by infiltration of CD8 T cells, which could also serve as an indicator of patient outcomes.^31^ Obstruction of the common bile duct is commonly observed in PC patients, leading to bile deficiency in the GI tract.^85^ Furthermore, mounting evidence suggests a correlation between the genus *Streptococcus* and liver cirrhosis, where metabolism and biosynthesis of bile acids and lipids are disrupted.^86^^,^^87^ This correlation highlights the prognostic and diagnostic potential of Streptococcus in distinguishing between obstructed and non-obstructed PHC as well as detecting liver metastasis in PC.^53^^,^^67^ Intriguingly, *Streptococcus* has shown the ability to differentiate PC based on tumor location, possibly with biliary obstruction being more common in PHC than in PBTC.^67^^,^^88^ Clinical outcomes, including patient prognosis, might be influenced by preoperative treatments that affect microbiome compositions, particularly the biliary microbiome, which may increase the chances of being influenced by the biological effects of *Enterococcus* spp. and *K*. *pneumoniae*.^58^^,^^69^^,^^89^ The pivotal aspect of these alterations pertains to treatment efficacy, especially in cases of medical-device-associated contamination where bacteria harboring cytidine deaminase (CDD) may deactivate gemcitabine.^72^ Given the presumed inevitability of this transmission, the strategic use of quinolones as an adjuvant targeted antibiotic therapy to modulate the effects of such bacteria, such as *K*. *pneumoniae*, is of great importance for improving treatment responses.^69^^,^^72^

Gut microbiota metabolites play a critical role as mediators in communication between the microbiota and host physiological processes. These bioactive compounds exert influence both locally within the gut and systemically by entering the bloodstream, making them promising targets for therapeutic intervention and disease diagnosis. Notably, TMAO has emerged as a significant metabolite implicated in both risk assessment and early detection of PC.^66^ TMAO levels in pancreatic cyst fluid indirectly reflect the composition of microbiota, showing positive correlations with bacteria such as *Granulicatella*, *Klebsiella*, *Stenotrophomonas*, *Streptococcus*, *Haemophilus*, and *Fusobacterium*, which were found to be elevated in IPMN and PC patients.^28^^,^^29^^,^^43^^,^^44^^,^^49^^,^^50^^,^^52^^,^^53^^,^^58^^,^^59^^,^^67^^,^^68^^,^^90^ Although TMAO shows promise as a biomarker and therapeutic target in PC, it is also implicated in various malignancies, including its potential correlation with aggressive forms of head and neck cancer, oral cancer, lung cancer, prostate cancer, and CRC.^91^ Moreover, the tryptophan metabolite 3-IAA has been investigated as a potential biomarker for chemotherapy response in PDAC, where its production has been associated with *B*. *fragilis* and *B*. *thetaiotaomicron*.^64^ Additionally, certain species such as short-chain fatty acid (SCFA)-producing bacteria like *F*. *prausnitzii* and *E*. *rectale*, as well as next-generation probiotics like *A*. *muciniphila*, have shown enrichment in the gut microbiota of PC-recovered patients.^27^^,^^52^^,^^60^^,^^68^ These findings suggest potential interventions through probiotic supplementation or dietary adjustments to improve disease outcomes in the future. However, caution is warranted due to the complex nature of microbiota interactions; for instance, *Akkermansia* may exhibit varying behaviors in different disease conditions.^92^ Therefore, a personalized approach is essential in navigating these complexities. In summary, this systematic review provides a comprehensive overview of microbial-related biomarkers from various sites (oral, gut, duodenum, bile, tissue, and blood) and their associations with clinical outcomes such as risk factor assessment, early detection, prognosis, and treatment efficacy. Additionally, it has catapulted interest in the evidence supporting the use of these markers in predicting and assessing the risk, diagnosis, and prognosis of PC. Based on current data, there is promising evidence that microbiome alterations from gut, oral cavity, or blood specimens may be used to develop new, non-invasive, and cost-effective tests that could potentially serve as alternatives to existing invasive PC screening tools like endoscopic ultrasound (EUS) in the future. Notably, combining these markers with other screening tests, such as serum CA-19-9, could improve diagnostic accuracy. However, most prediction models are derived from case-control studies and require further validation through high-quality prospective research. Future studies should prioritize creating standardized and reproducible protocols for human microbiome analysis to enable better comparability and more robust conclusions. Practical considerations, such as cost-effectiveness, affordability, and acceptance by patients and healthcare providers, must also be evaluated before microbiome analysis can be widely adopted for PC screening. Taken together, these research advancements provide a unique opportunity to implement microbiota discoveries into clinical applications, including prevention, early detection, and treatment.

### Limitations of the study

In our study, we acknowledge the limitation imposed by the diverse methodologies used in microbiome analysis, which can result in inconsistent and sometimes conflicting results. To tackle this challenge, we meticulously extracted comprehensive information regarding the methodologies employed in the studies included in our review. By doing so, we aimed to provide a clearer understanding of how variations in techniques and analysis approaches may influence the findings. This rigorous approach will help identify key methodological factors that contribute to discrepancies, paving the way for more standardized protocols and facilitating more robust comparisons across studies.

## Resource availability

### Lead contact

Requests for further information and resources should be directed to and will be fulfilled by the lead contact, Hamidreza Houri (hr.houri@sbmu.ac.ir).

### Materials availability

This study did not generate new unique reagents.

### Data and code availability

• Data: no data was used for the research described in the article.
• Code: this paper did not report original code.
• Any additional information is available from the lead contact upon request.

## Acknowledgments

This study is related to the project NO. 1403/41749 from Student Research Committee, Shahid Beheshti University of Medical Sciences, Tehran, Iran. The authors appreciate the “Student Research Committee” and “Research & Technology Chancellor” in 10.13039/501100005851Shahid Beheshti University of Medical Sciences for their financial support of this study. The authors also wish to extend their sincere appreciation to the members of the Research Institute for Gastroenterology and Liver Diseases affiliated with 10.13039/501100005851Shahid Beheshti University of Medical Sciences, for their invaluable cooperation and support during the execution of this study.

Financial support for this study was provided by the Student Research Committee affiliated with 10.13039/501100005851Shahid Beheshti University of Medical Sciences, Tehran, Iran under grant number 1403/41749.

## Author contributions

Z.H. contributed to conceptualization, literature review, data extraction, and writing the original draft. F.S. was involved in conceptualization, data extraction, and writing review and editing. S.A. participated in the literature review, data extraction, and writing review and editing. B.K. handled description analysis. A.S. contributed to conceptualization, supervision, and review and editing. N.A. and V.P. participated in the literature review and writing review. H.H. was responsible for conceptualization, supervision, and writing review and editing. All authors reviewed and approved the final manuscript and take responsibility for the integrity and accuracy of the work, ensuring any questions related to the study are thoroughly addressed.

## Declaration of interests

The authors declare no competing interests.

## STAR★Methods

### Key resources table

**Table:** 

REAGENT or RESOURCE	SOURCE	IDENTIFIER
**Software and algorithms**		

Microsoft Excel	Microsoft	N/A
EndNote	Clarivate Analytics	N/A

### Method details

#### Study design

This study was conducted following the Preferred Reporting Items for Systematic Reviews and Meta-Analyses (PRISMA) guidelines.^93^ The study protocol was registered in the International Prospective Register of Systematic Reviews (Registeration code: CRD42023426485).

#### Search strategy and screening

We comprehensively conducted a systematic search in MEDLINE (via PubMed), Web of Science, and EMBASE databases for articles published between 2001 and 2024. A combination of following syntax was considered: “*pancreatic neoplasms*” MeSH Terms (including “pancreatic neoplasms” “pancreatic cancer” [Title/Abstract] OR “pancreatic ductal adenocarcinoma”[Title/Abstract] OR “pancreatic neoplasm”[Title/Abstract] OR “intraductal papillary mucinous neoplasm”[Title/Abstract] OR “acinar cell carcinoma”[Title/Abstract]) AND “*microbiota*” MeSH Terms (including “microbiota”[Title/Abstract] OR “microbiotas”[Title/Abstract] OR “microbiotas”[Title/Abstract] OR “microbiotae”[Title/Abstract] OR “microbiome” [Title/Abstract]). The search strategy details for each database are presented in Table S1. Titles, abstracts, and full texts were independently screened by three authors (Z.H., S.A., and F.S.) based on predefined selection criteria. Any disagreements were reviewed and resolved through discussion with a senior investigator (H.H.). Additionally, a cross-referencing of eligible studies was performed to identify any potentially ignored research contributions.

#### Eligibility criteria

Studies were included if they met the following eligibility criteria: (i) observational studies exploring the links between microbiota and pancreatic neoplasia, with a particular focus on microbial biomarkers used in screening, diagnosis, prognosis, and treatment efficacy; (ii) studies developing prediction models for early detection and prognosis of pancreatic neoplasia based on microbial biomarkers. The exclusion criteria were as follows: (i) studies conducted on insufficient sample size (fewer than 8 individuals for each group) due to their limited statistical power and the unreliability of findings; (ii) studies lacking precise biomarkers of interest or failing to mention any associations; (iii) *in-silico*, *in-vitro*, and *in-vivo* experiments; (iv) studies with no separation of pancreatic neoplasia from other cancers; (v) studies with no full-text availability.

#### Data extraction

For each eligible study, the following data were extracted: year of publication, study country, population country, study design, number of cases and controls, clinical outcomes (including screening or risk factor, early detection and diagnosis, prognosis, and treatment efficacy), sample type, chemotherapy treatment, and methodology. Differences in alpha and beta diversity, alterations in bacterial or fungal taxa at various taxonomic levels (phylum, class, order, family, genus, or species), and any statistical measures of association (such as odds ratio (OR), hazard ratio (HR), fold change, and linear discriminative analysis (LDA) score) relative to any microbial biomarker (e.g., abundance, microbiota-derived metabolites) were extracted and reported as the main results. These analyses provide a comprehensive overview of the microbial community structure and its associations with specific biomarkers. Furthermore, in order to organize the data, three layers of information extraction were employed, and studies were evaluated based on these layers. The first layer of data was related to microbial abundance alterations based on linear discriminant analysis Effect Size (LefSe) scores. The second layer of data was considered for any statistical metrics in detail, such as HR, OR, fold change, follow-up data, study type, matched factors, along with microbial biomarkers related to each statistical measure. Finally, the third layer of data included the studies with prediction models extracting data like microbial predictors of models, matched factors, sensitivity, specificity, area under the curve (AUC), internal and external validation information.

#### Quality assessment

The studies included in this systematic review were evaluated for quality and methodological reliability using the Newcastle-Ottawa Scale (NOS), which is specifically designed for assessing observational studies.^94^ This scale comprises eight components that assess the selection of study participants, the comparability of study groups, and the clarity and accuracy of reported outcomes. Studies scoring 8-9 and 6-7 were considered very good and good studies, respectively, while those scoring below 5 were classified as unsatisfactory studies and thus excluded from our study (Table S2). Given the considerable heterogeneities among the studies included, quantitative analysis was unattainable within the parameters of this study. Consequently, we carried out descriptive syntheses and presented the extracted data in a narrative and thematic manner to enhance understanding and facilitate interpretation. Furthermore, for studies involving prediction models, we assessed each model utilizing the Checklist for Critical Appraisal and Data Extraction of Systematic Reviews of Prediction Modelling Studies (CHARMS).^95^ This checklist involved five main domains: participant selection, predictor reporting, loss to follow-up, clinical outcome, and data analysis (Table S3).

### Quantification and statistical analysis

This study did not involve any statistical analyses.

## Supplemental information

Document S1. Tables S1–S3

Data S1. Detailed information of studies based on reported microbial abundance at different levels, diversity, and methods Supplemental tables containing Tables S1–S3.
