# Supplementary material for: Microbiota as a state-of-the-art approach in precision medicine for pancreatic cancer management: A comprehensive systematic review
Source: iScience. 2025 Mar 28;28(5):112314. doi: 10.1016/j.isci.2025.112314 (PMC12019022; doi:10.1016/j.isci.2025.112314)
Supplement: Document S1. Tables S1–S3 [file mmc1.pdf]

iScience, Volume *28*

Supplemental information

Microbiota as a state-of-the-art approach in precision medicine for pancreatic cancer management: A comprehensive systematic review

Zeinab Hesami, Fattaneh Sabzehali, Babak Khorsand, Samira Alipour, Amir Sadeghi, Nastaran Asri, Valerio Pazienza, and Hamidreza Houri

Microbiota as a State-of-the-art Approach in Precision Medicine for Pancreatic Cancer Management: A Comprehensive Systematic Review

Supplemental Tables

Table S1. Search strategy used among different databases.

**Search strategy**	**Year**
(“pancreatic neoplasms”[mesh] OR Pancreatic Neoplasm*[tiab] OR Pancreas Neoplasm[tiab] OR Cancer of Pancreas[tiab] OR Pancreas Cancers[tiab] OR Cancer of the Pancreas[tiab] OR Pancreas Cancer[tiab] OR Pancreatic Cancer[tiab] OR Cancers, Pancreatic[tiab] OR Pancreatic Cancers[tiab] OR Pancreatic Carcinoma[tiab] OR Pancreatic Carcinomas[tiab] OR Pancreatic Acinar Carcinoma[tiab] OR Pancreatic Acinar Carcinomas[tiab]) AND (“Microbiota”[mesh] OR Microbiotas[tiab] OR Microbial Community[tiab] OR Microbial Communities[tiab] OR Microbial Community Composition*[tiab] OR Microbiome*[tiab] OR Human Microbiome*[tiab] OR Microbial Community Structure*)	2013-2024
(‘pancreas tumor’/exp OR ‘neoplasia of the pancreas’:ab,ti OR ‘neoplasm of the pancreas‘:ab,ti OR ‘neoplastic pancreas‘:ab,ti OR ‘neoplastic pancreatic’:ab,ti OR ‘pancreas neoplasia‘:ab,ti OR ‘pancreas neoplasm‘:ab,ti OR ‘pancreas tumorigenesis‘:ab,ti OR ‘pancreas tumour‘:ab,ti OR ‘pancreatic neoplasm‘:ab,ti OR ‘pancreatic tumor‘:ab,ti OR ‘pancreatic tumorigenesis’:ab,ti OR ‘pancreatic tumour’:ab,ti OR ‘tumor of the pancreas’:ab,ti OR ‘tumour of the pancreas’:ab,ti OR ‘pancreas tumor’:ab,ti) AND (‘microflora’/exp OR ‘microbial flora’:ab,ti OR ‘microbiota‘:ab,ti OR ‘microflora ‘:ab,ti)	2000-2024
(TS= (“Pancreatic Neoplasm” OR “Pancreas Neoplasm*” OR “Cancer of Pancreas” OR “Pancreas Cancers” OR “Cancer of the Pancreas” OR “Pancreas Cancer” OR “Pancreatic Cancer” OR “Pancreatic Cancers” OR “Pancreatic Carcinoma” OR “Pancreatic Carcinomas” OR “Pancreatic Acinar Carcinoma” OR “Pancreatic Acinar Carcinomas” OR “neoplasia of the pancreas” OR “neoplasm of the pancreas” OR “neoplastic pancreas” OR “neoplastic pancreatic” OR “pancreas neoplasia” OR “pancreas neoplasm” OR “pancreas tumorigenesis” OR “pancreas tumour” OR “pancreatic neoplasm” OR “pancreatic neoplasms” OR “pancreatic tumor” OR “pancreatic tumorigenesis” OR “pancreatic tumour” OR “tumor of the pancreas” OR “tumour of the pancreas” OR “pancreas tumor”)) AND (TS= (“Microbiotas” OR “Microbial Communit*” OR “Microbial Community Composition” OR “Microbial Community Compositions” OR “Microbiome*” OR “Human Microbiome*” OR “Microbial Community Structure” OR “Microbial Community Structures” OR “microbial flora” OR “microbiota” OR “microflora”))	2011-2024

Table S2. Quality assessment of the 41 eligible studies using the Newcastle-Ottawa Scale (NOS) tool.

**Case-Control studies**	Selection				Comparability	Exposure			Total score
Author, year	Is the case definition adequate?	Representativeness of the cases	Selection of controls	Definition of controls	Comparability of cases and controls on basis of the design or analysis?	Ascertainment of exposure	Same method of ascertainment for cases and controls	Adjustment for multiple testing	
Farrell, 2011	1	1	1	1	2	1	1	1	9
Alkharaan, 2020	1	1	0	1	1	1	1	0	6
Guo, 2021	1	1	0	0	2	1	1	0	6
Kartal, 2022	1	1	0	1	2	1	1	1	8
Chen, 2023	1	1	1	1	2	1	1	1	9
Half, 2019	1	1	1	1	2	1	1	1	9
Torres, 2015	1	1	1	1	1	1	1	0	7
Lu, 2019	1	1	1	1	2	1	1	1	9
Vogtmann, 2020	1	1	0	1	1	1	1	1	7
Wei, 2020	1	1	1	1	2	1	1	1	9
Kim, 2021	1	1	1	1	2	1	1	1	9
Zhou, 2021	1	1	1	1	2	1	1	1	9
Kohi, 2022	1	1	0	1	1	1	1	1	7
Negata, 2022	1	1	0	1	2	1	1	1	8
Ren, 2017	1	1	1	1	2	1	1	1	9
Gaiser, 2019	1	1	0	1	2	1	1	0	7
Del Castillo, 2019	1	1	0	1	2	1	1	1	8
Guo, 2022	1	1	0	0	2	1	1	1	7
Sun, 2020	1	1	1	1	1	1	1	1	8
Risch, 2014	1	1	0	1	1	1	1	1	7
Jeong, 2020	1	1	0	0	2	1	1	0	6
Hozaka, 2023	1	1	0	1	1	1	1	0	6
Li, 2022	1	1	0	1	2	1	1	1	8
Vietsch, 2023	1	1	0	1	2	1	1	1	8
Irajizad, 2023	1	1	1	1	2	1	1	1	9
**Cohort studies**	Selection				Comparability	Outcome			Total score

Author, year	Representativeness of the cases	Selection of the control	Ascertainment of exposure	Primary diagnosed at start of study	Comparability of cohorts on basis of the design or analysis?	Ascertainment of outcome	Was follow-up long enough for outcomes to occur?	Adequacy of follow-up of cohorts	
Fan, 2017	1	0	1	0	2	1	1	1	7
Riquelme, 2019	1	1	1	1	2	1	1	1	9
Negata, 2022	1	1	1	1	2	1	1	1	9
Yang, 2023	1	0	1	0	2	1	1	0	6
Matsukawa, 2021	1	0	1	1	2	1	1	1	8
Huang, 2020	1	0	1	0	1	1	1	1	6
Guenther, 2022	1	1	1	1	2	1	1	1	9
Kharofa, 2023	1	1	1	1	1	1	1	1	8
Mitsuhashi, 2015	1	1	1	1	1	1	1	1	8
Weniger, 2021	1	1	1	1	1	1	1	1	8
Stolzenberg-Solomon, 2001	1	0	1	0	1	1	1	1	6
Michaud, 2013	1	0	1	0	1	1	1	1	6
Petrick, 2022	1	0	1	0	2	1	1	1	7
Yu, 2013	1	0	1	0	1	1	1	1	6
Tintelnot, 2023	1	1	1	1	2	1	1	1	9
Abe, 2024	1	1	1	0	2	1	1	1	8
Stein-Thoeringer, 2024	1	1	1	1	1	1	1	1	8
Irajizad, 2023	1	1	1	1	2	1	1	1	9

Table S3. Risk of bias assessment for 13 studies based on CHARMS checklist.

**Studies developing prediction models**	**Domain**				
Author, year	Participants	Predictor	Outcome	Attrition	Analysis
Farrell, 2011	Low	Low	Low	High	High
Riquelme, 2019	Low	Low	Low	Low	Medium
Kartal, 2022	Low	Low	Low	Medium	Low
Chen, 2023	Low	Low	Low	High	Medium
Half, 2019	Low	Low	Low	High	Low
Lu, 2019	Low	Low	Low	High	Medium
Kim, 2021	Low	Low	Low	High	Medium
Zhou, 2021	Low	Low	Low	High	Medium
Negata, 2022	Low	Low	Low	Low	Low
Yang, 2023	Low	Low	Low	Low	High
Ren, 2017	Low	Low	Low	High	Medium
Li, 2022	Low	Low	Low	High	High
Irajizad, 2023	Low	Low	Low	Low	Low
